# DON‐Loaded Nanodrug‐T Cell Conjugates With PD‐L1 Blockade for Solid Tumor Therapy

**DOI:** 10.1002/advs.202501815

**Published:** 2025-04-24

**Authors:** Xin Yang, Xiaoshuang Niu, Ye Su, Xiaoyun Ye, Wanqiong Li, Wenxuan Zeng, Xin Zhao, Zhuoying He, Qingyu Dong, Xiuman Zhou, Xinghua Sui, Guanyu Chen, Yanfeng Gao, Juan Liu

**Affiliations:** ^1^ School of Pharmaceutical Sciences (Shenzhen) Shenzhen Campus of Sun Yat‐sen University Shenzhen 518107 China

**Keywords:** Adoptive T‐cell therapy, Cancer immunotherapy, Glutamine metabolism, Solid tumor, T cell‐nanodrug conjugate

## Abstract

Adoptive T‐cell therapy (ACT) holds significant promise for treating solid tumors but is often constrained by insufficient T‐cell infiltration, survival, and functional persistence. To overcome these obstacles, we developed DON‐loaded nanodrug‐T cell conjugates with PD‐L1 blockade, forging a dynamic mutualistic relationship between T cells and therapeutic agents. Sustained release of glutamine antagonist 6‐diazo‐5‐oxo‐L‐norleucine (DON) within these conjugates continuously enhances T‐cell endurance and potency by promoting memory differentiation and elevating crucial adhesion and motility genes. Concurrently, PD‐L1 blocking peptides liberate T cells from immunosuppression, assisting T cells with precision toward tumor sites. This dual‐targeting strategy—T cells directed at tumor antigens and peptides at PD‐L1— enriches the tumor microenvironment with potent therapeutics, amplifying T cell‐driven tumor destruction. Our approach effectively overcomes the critical barriers of ACT—infiltration, persistence, and efficacy—unlocking the full therapeutic potential of T‐cell therapy against complex solid tumors.

## Graphical abstract

This study establishes DON‐loaded T cell‐nanodrug conjugates with PD‐L1 blockade, creating a synergistic system. As a glutamine antagonist, sustained DON release enhances T‐cell endurance and infiltration by promoting memory differentiation and upregulating adhesion and motility genes, while PD‐L1 blockade alleviates immunosuppression and directs T cells to tumors, together unlocking the full potential of T‐cell therapy for solid tumors.

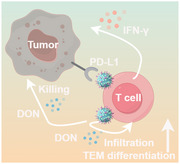

## 1 Introduction

Adoptive T‐cell therapy (ACT) has effectively treated hematologic malignancies but faces significant challenges when applied to solid tumors, underscoring the necessity for advanced therapeutic strategies.^[^ ^1^ ^]^ Solid tumors are notoriously difficult to treat due to their dense extracellular matrix and immunosuppressive microenvironment, which impede T‐cell infiltration, survival, and function, thereby reducing the effectiveness of traditional ACT.^[^ ^2^ ^]^ To overcome these challenges, the concept of mutualism—a symbiotic relationship where both species benefit—has been ingeniously applied in synthetic biology through the development of cell‐drug conjugates. These conjugates exemplify mutualistic interactions between T cells and therapeutic agents, with each component enhancing the effectiveness of the other, creating a potent and synergistic therapeutic platform specifically designed for the complexities of solid tumors.^[^ ^3^ ^]^

The foundation of this innovative approach lies in the application of chemical engineering, which facilitates the integration of multiple therapeutic agents within a single cellular platform.^[^ ^4^ ^]^ This integration enables the design of combination therapies that concurrently target a range of biological pathways, thereby modulating T cell functionality and reconfiguring the tumor microenvironment.^[^ ^5^ ^]^ Chemically engineered cells serve as precise delivery vehicles, ensuring that therapeutic agents are concentrated at the tumor site, thereby minimizing off‐target effects.^[^ ^6^ ^]^ Unlike genetic engineering which imposes lasting genome alterations, chemical engineering offers the distinct advantage of transient modifications.^[^ ^7^ ^]^ These temporary adjustments are confined to individual cells, thereby mitigating the long‐term risks associated with irreversible genetic changes.^[^ ^8^ ^]^

One approach involves engineering T cells with TCR signaling‐responsive IL‐15 nanogels for treating B16F10 melanoma. These nanogels release IL‐15 upon membrane reduction, enhancing T‐cell activity and demonstrating superior efficacy compared to T cells with free IL‐15.^[^ ^9^ ^]^ However, since IL‐15 release is only triggered by T‐cell activation, the time to fully induce beneficial T‐cell phenotype changes is limited. Another strategy uses liposome‐based T cell‐nanodrug conjugates to deliver avasimibe, which boosts T cell vitality by increasing membrane cholesterol levels.^[^ ^10^ ^]^ Yet, unclear drug release dynamics from the liposome create uncertainty in T cell modulation. These previous designs highlight the substantial potential of T cell‐nanodrug conjugates. Achieving effective solid tumor treatment requires comprehensive strategies that integrate the regulation of T cell phenotype, functionality, infiltration, and motility. Furthermore, while current approaches primarily focus on enhancing T cell functionality, they often neglect the direct tumor‐killing potential of the therapeutic agents, which is crucial for reducing tumor burden and improving overall outcomes.

6‐Diazo‐5‐oxo‐L‐norleucine (DON) is a glutamine antagonist that inhibits glutamine metabolism. Recent studies have highlighted the potential of disrupting glutamine metabolism with DON to promote the differentiation of T cells into long‐lived, activated memory phenotypes through oxidative metabolism.^[^ ^11^ ^]^ *Ex vivo* pre‐treatment of CAR‐T cells with DON has shown promise in enhancing their preparation efficiency and antitumor efficacy.^[^ ^12^ ^]^ Additionally, DON has demonstrated efficacy in killing tumor cells by targeting glutamine metabolism.^[^ ^13^ ^]^ However, its systemic toxicity limits its clinical use, underscoring the need for targeted delivery systems that maximize therapeutic benefits while minimizing risks.^[^ ^14^ ^]^

**Figure 1:**
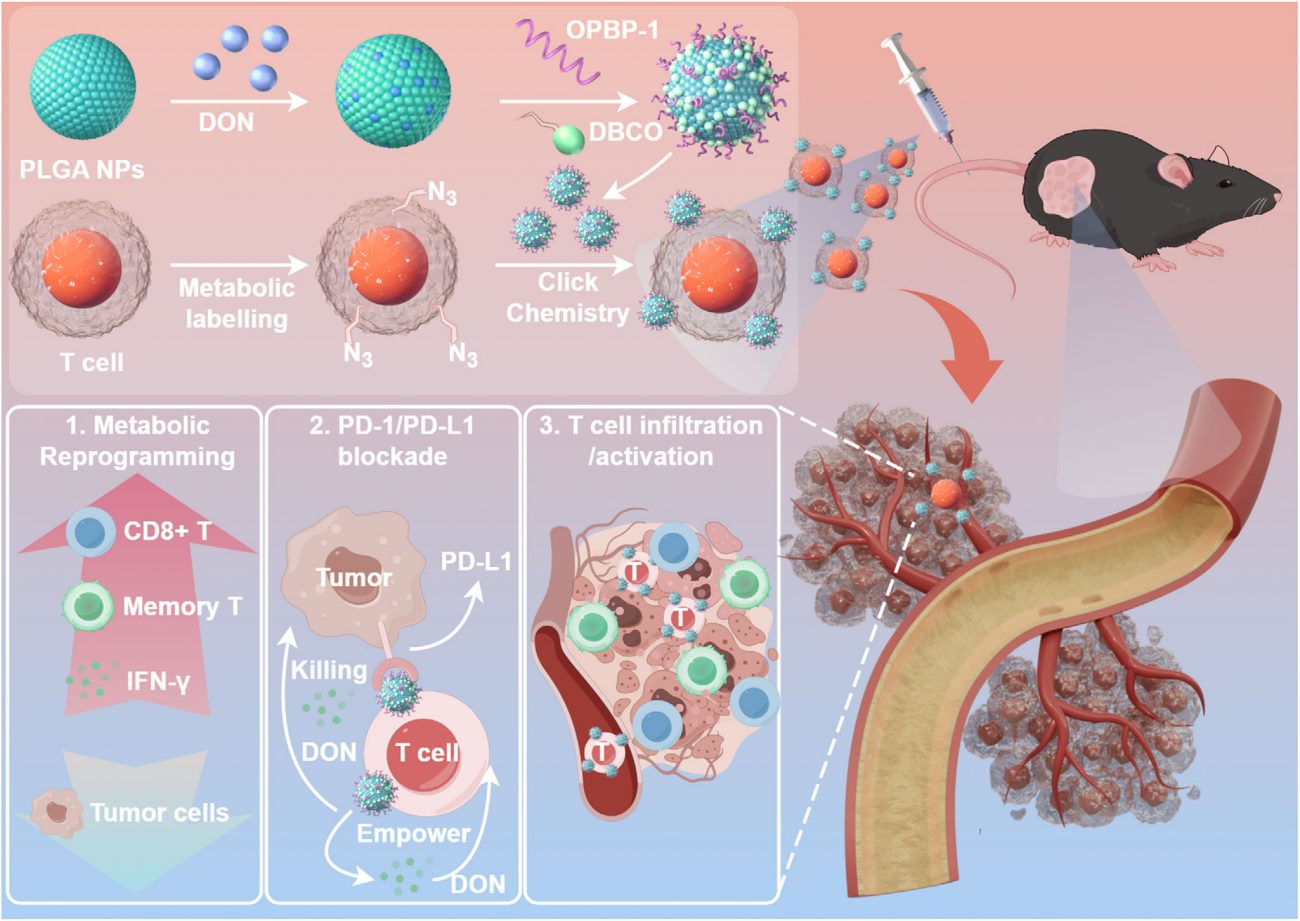
Schematic illustration of T Cell‐nanodrug conjugates enhancing ACT against solid tumors. DBCO‐functionalized OPBP‐1‐PLGA‐DON nanodrugs are anchored to azido‐bearing OT‐1 T cells via click chemistry. Upon intravenous administration in mice, these conjugates sustainably release DON, driving memory T‐cell differentiation, infiltration, and tumor eradication. The surface‐conjugated PD‐L1 blocking peptide OPBP‐1 disrupts PD‐1/PD‐L1 interactions between T cells, tumor cells, and dendritic cells, thereby liberating T‐cell activity and enhancing accumulation within PD‐L1‐expressing tumors. OT‐1 T cells, acting as precise delivery vehicles for DON, execute tumor cell killing while mitigating systemic toxicity. This synergistic combination significantly boosts ACT efficacy against solid tumors. The figure was generated using Figdraw.

To address these challenges, we aim to develop poly (lactic‐*co*‐glycolic acid) (PLGA) nanoparticles incorporating DON and PD‐L1 blocking peptide OPBP‐1, creating T Cell‐nanodrug conjugates (OPBP‐1‐PLGA‐DON‐T cells). This approach involves a biorthogonal reaction between dibenzocyclooctyne (DBCO)‐functionalized OPBP‐1‐PLGA‐DON nanodrugs and metabolically labeled azido‐bearing T cells. Biocompatible PLGA nanoparticles enable sustained DON release, continuously modulating T cells. Surface‐conjugated PD‐L1 blocking peptides liberate T cells from PD‐1/PD‐L1 interactions and promote T‐cell accumulation within PD‐L1‐expressing tumors, amplifying efficacy and reducing off‐target toxicity. The spatially coordinated deployment of DON, facilitated by PD‐L1 blocking peptides and T‐cell targeting, culminates in the targeted enrichment of this therapeutic combination within tumors. By leveraging cell‐drug conjugates that combine T cells, DON‐loaded nanodrugs, and PD‐L1 blocking peptides, this strategy aims to overcome fundamental challenges of ACT—infiltration, persistence, efficacy, and safety—thereby enhancing the effectiveness of T‐cell therapies against solid tumors (**Figure** **1**).

## 2 Results and Discussion

### 2.1 Effects of DON on T‐cell Function and Migration

**Figure 2:**
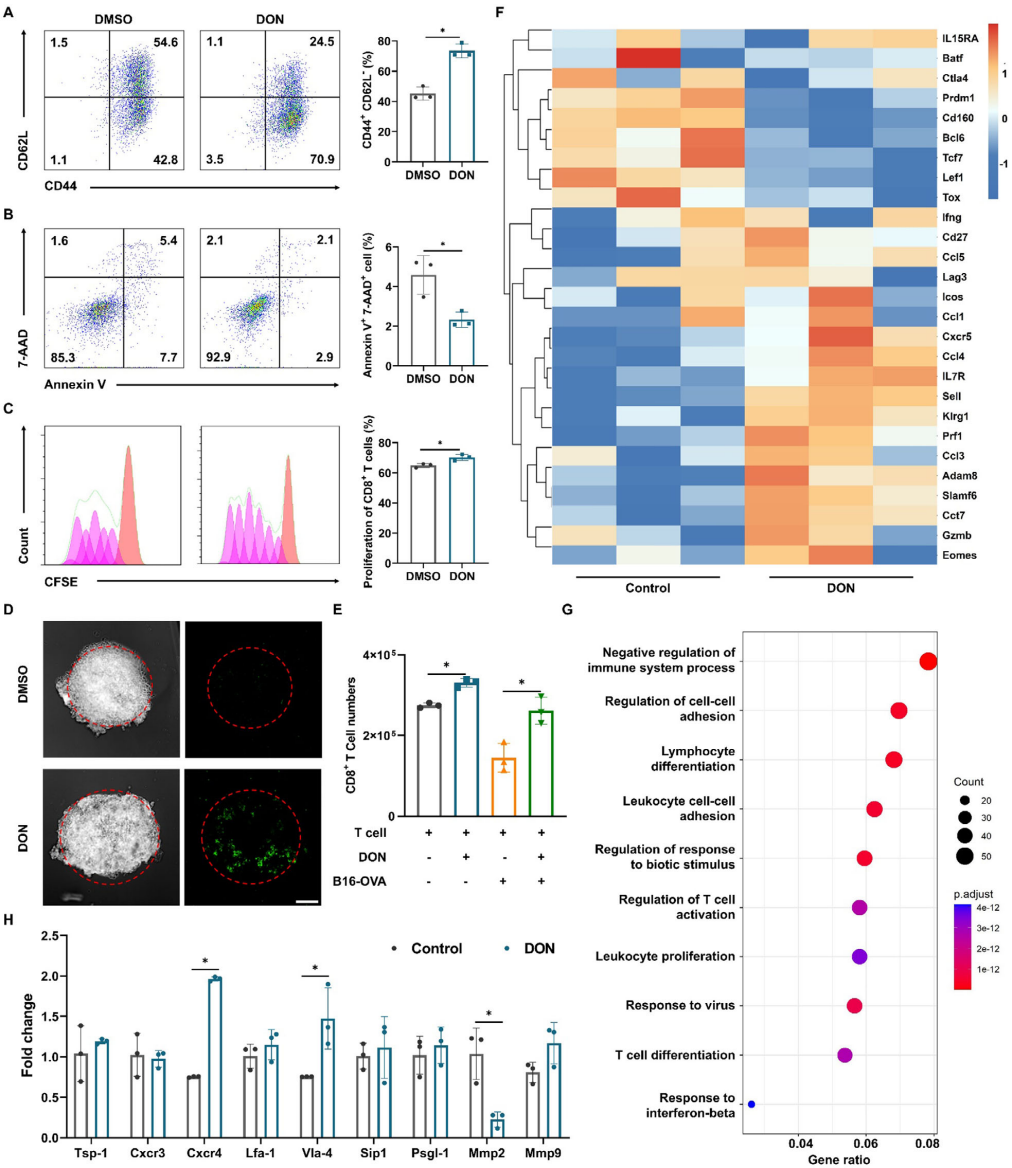
In vitro functional validation of DON. Effects of DON on the differentiation (A) and the apoptosis of CD8^+^ T cells (B). T cell memory differentiation was analyzed by flow cytometry using CD44 and CD62L markers following 0.3 µM DON treatment for 72 h. Apoptosis was assessed after 5 d of DON treatment using Annexin V and 7‐AAD staining and analyzed by flow cytometry. (C) Proliferative activity of CD8^+^ T cells treated with DON assessed via CFSE assay. The orange peak indicates the initial generation, with peaks to the left representing subsequent proliferative generations. Untreated cells show five generations of proliferation (left), while DON‐treated cells display six generations (right), indicating enhanced proliferation. (D) Infiltration of CFSE‐stained CD8^+^ T cells into 3D tumor spheroids after 0.3 µM DON treatment for 24 h (green indicates CFSE‐stained CD8^+^ T cells; scale bar: 200 µm). (E) The number of CD8^+^ T cells migrating from the upper chamber to the lower chamber after treating with DON. (F) Heatmap showing differentially expressed genes in CD8^+^ T cells treated with DON in vitro for 3 days. (G) Pathway enrichment analysis of differentially expressed genes in CD8^+^ T cells treated with DON in vitro for 3 days. (H) qPCR detection of the effect of DON on the expression of mouse CD8^+^ T cell motility genes. n = 3, statistical significance was calculated using unpaired, one‐sided Mann‐Whitney test. ^*^ *P* < 0.05.

We investigated DON's role as a metabolic checkpoint inhibitor in regulating T‐cell functions. Similar to what Rober and others found,^[^ ^15^ ^]^ our initial findings revealed a robust suppression of cell proliferation in the B16‐OVA tumor cell line following DON‐induced inhibition of glutamine metabolism, while murine‐derived T cells remained unaffected, suggesting a selective cytotoxic effect (Figure S1A,B). To further investigate this phenomenon, flow cytometry was employed to analyze the expression of the memory markers CD44 and CD62L, elucidating the effects of the glutamine inhibitor DON on T cell phenotypes. Our results indicate that when glutamine metabolism is inhibited, T cells undergo metabolic compensation to develop into memory T cells (**Figure** **2**A), accompanied by a noticeable decrease in apoptotic susceptibility (Figure 2B). Moreover, dye dilution experiments using carboxyfluorescein succinimidyl ester (CFSE) demonstrated that DON‐mediated glutamine inhibition maintained a stronger proliferation capacity in T cells (Figure 2C).

Following our initial investigations, we explored the impact of DON treatment on T cell migration and infiltration capabilities, which had not been previously illustrated. Co‐incubation of T cells with 3D tumor spheroids demonstrated a significant enhancement in the infiltration of CFSE‐labeled T cells into the interior of tumor spheroids, indicating an augmented migratory response (Figure 2D and S2). To further validate the migratory response of T cells to DON, a transwell assay was conducted. T cells were placed in the upper wells, while DON, with or without tumor cells, was placed in the lower wells. The results showed a marked increase in T cell migration to the lower well in the presence of DON, regardless of tumor cell presence. These findings underscore DON's role in enhancing T cell motility (Figure 2E).

Transcriptomic analysis of DON‐treated T cells reveals significant increases in the expression of migration‐related genes like *Ccl3* and *Cxcr5*, as well as effector genes in T cells such as *Gzmb* and *Ifng*, and stemness‐related genes like *Tcf‐*7 and *Ccr7*. Conversely, exhaustion‐related genes like *Tox* and *Lag3* exhibited significant decreases (Figure 2F). Additionally, the pathway enrichment result reveals significant enrichment of genes associated with cell adhesion, migration, and differentiation (Figure 2G). The volcano plot of differentially expressed genes also indicated significant upregulation of genes involved in regulating cell adhesion, such as *Cdh1* and *Rgs16* (Figure S3A). Moreover, qPCR examination of T cells treated with DON revealed significant changes in the expression levels of motility‐related genes such as *Cxcr4* and *Vla‐4*. Notably, stemness genes including *Tcf1* and *Ccr7* displayed increased expression levels, while apoptosis and exhaustion‐related genes like *Tox* and *Bcl6* exhibited significant decreases (Figure 2H, S3B). These cumulative findings suggest that DON serves as a pivotal metabolic regulator, fostering the differentiation of T cells into a memory phenotype characterized by augmented self‐renewal capacities and prolonged longevity. Furthermore, DON enhances T cell motility and adhesion, facilitating their infiltration into solid tumors, a critical aspect of effective solid tumor therapy. Therefore, through a thorough investigation of DON‐T cell interactions, we confirm that DON is an ideal candidate for constructing T cell‐drug conjugates against tumors.

### 2.2 Construction and Characterization of OPBP‐1‐PLGA‐DON‐T Cells

**Figure 3:**
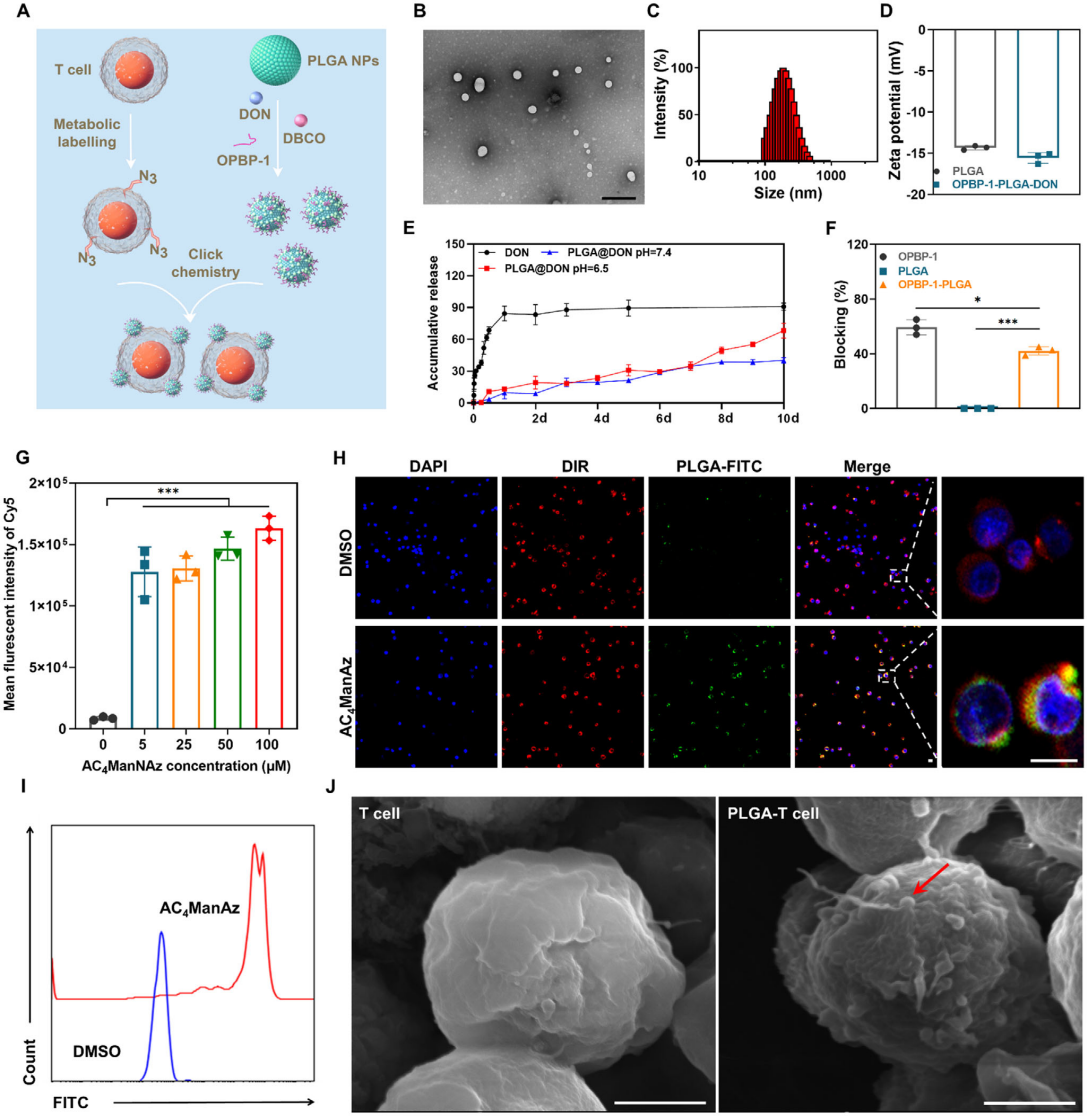
Construction and characterization of OPBP‐1‐PLGA‐DON‐T cells. The figure was generated using Figdraw. (A) Schematic representation of the design and the preparation of OPBP‐1‐PLGA‐DON. (B) Transmission electron microscopy (TEM) images displaying the morphology of OPBP‐1‐PLGA‐DON. (C) The size distribution of OPBP‐1‐PLGA‐DON was measured using dynamic light scattering (DLS) with a Malvern particle size analyzer. (D) The surface potential of OPBP‐1‐PLGA‐DON illustrates their electrostatic properties. (E) Drug release profiles of OPBP‐1‐PLGA‐DON under physiological and tumor‐mimicking conditions (pH 7.4 and 6.5), demonstrating sustained release behavior over 10 days. (F) Blocking activity of the PD‐L1 OPBP‐1 on PLGA nanoparticles assessed by flow cytometry. Fluorescence intensity reflects PD‐L1 binding to PD‐1. Blocking efficacy was quantified by comparing fluorescence reduction in nanoparticle‐treated samples relative to untreated controls. (G) Efficiency evaluation of azido‐glucose modification of T cell. (H) Visualization of OPBP‐1‐PLGA‐DON‐T cells using confocal microscopy, with blue representing cell nuclei, red indicating cell membranes, and green depicting FITC‐labeled PLGA nanoparticles. After incubating ordinary T cells or azide‐modified T cells with DBCO‐PLGA‐FITC at 37 °C for 1 h, the cells were examined using a fluorescence confocal microscope. In the conjugation group, the green nanoparticles were observed to co‐localize with red cell membranes, indicating successful conjugation of PLGA nanoparticles to the T cells. Scale bar: 15 µm. (I) The loading of OPBP‐1‐PLGA‐DON on T cells was characterized by flow cytometry. (J) Morphological features of T cell‐PLGA conjugates characterized by SEM. Scale bar: 2 µm, n = 3. Statistical significance was calculated using unpaired, one‐sided Mann‐Whitney test. ^***^ *P* < 0.001.

The T Cell‐nanodrug conjugates, designated as OPBP‐1‐PLGA‐DON‐T cells, were prepared as shown in **Figure** **3**A. First, PLGA nanoparticles loaded with DON were prepared to be approximately 200 ± 1.98 nm in size with a negative surface charge (Figure 3B‐D). Additionally, we assessed the DON content in the supernatant of the nanoparticle preparation using high‐performance liquid chromatography (HPLC) and calculated the drug loading and encapsulation efficiency based on the total amounts of DON and PLGA added. The calculated drug loading rate of PLGA was 4.1 ± 0.31%, and the encapsulation efficiency was 40 ± 2.13%. The drug release profile of the PLGA nanoparticles was evaluated under pH 7.4 and pH 6.5 conditions to mimic physiological and tumor microenvironments (Figure 3E). At pH 6.5, approximately 68.13 ± 5.77% of DON was released by day 10, confirming the sustained release capability of the nanoparticles, consistent with other studies on PLGA‐based drug delivery systems. The release results indicate that the nanoparticles exhibit a biphasic release profile: the initial phase for the first five days is characterized by diffusion‐dominated release that is pH‐independent. Subsequently, under pH 6.5 conditions, the release rate significantly increases due to the accelerated hydrolysis of PLGA. This pH‐dependent long‐term release variation is consistent with existing studies.^[^ ^16^ ^]^ Furthermore, OPBP‐1‐PLGA‐DON‐conjugated T cells were found to efficiently accumulate at tumor sites within 12 h (Figure 5D), during which approximately 3.8 ± 0.5% and 10.6 ± 0.5% of DON at both pH 7.4 and pH 6.5 was released, as estimated from the *ex vivo* release profile. This controlled release aligns with the timeline for T‐cell homing, reducing premature drug loss and ensuring targeted and effective drug delivery.

Secondly, PLGA nanoparticles were further modified with the PD‐L1 blocking peptide OPBP‐1 (Figure S4). Using fluorescence correlation spectroscopy (FCS), we quantified the number of peptides per nanoparticle by dividing the counts per molecule of nanoparticles by the counts per molecule of free Atto488‐labeled peptides, yielding an average of 202 ± 53 peptides per nanoparticle (Figure S5). To determine whether drug loss during the surface modification process was substantial, we performed quantitative analysis of DON content in the collected supernatants throughout the conjugation and washing processes. The results indicate that the DON loss ratio was 4.47 ± 0.3%, demonstrating that while a small amount of drug was lost, it remained minimal relative to the total drug loading. To evaluate the ability of OPBP‐1‐modified PLGA nanoparticles to inhibit the PD‐1/PD‐L1 interaction, CHO‐K1‐PD1 cells were treated with free PD‐L1 proteins in the presence or absence of the nanoparticles. Flow cytometry was used to measure the fluorescence intensity, reflecting the extent of PD‐L1 binding to PD‐1. Untreated cells served as a positive control for maximum PD‐L1 binding, while cells without PD‐L1 treatment were used to determine background fluorescence. The blocking efficacy of the OPBP‐1‐modified PLGA nanoparticles was quantified to be 42.17 ± 2.48% by comparing the reduction in fluorescence intensity in nanoparticle‐treated samples relative to the positive control, providing a clear measure of their ability to disrupt the PD‐1/PD‐L1 interaction (Figure 3F).

For conjugating OPBP‐1‐PLGA‐DON onto T cells, we isolated lymphocytes from the spleens and lymph nodes of C57BL/6 mice, and CD8^+^ T cells were purified using magnetic bead separation. Metabolic cell‐labeling technology was employed to label T cells with azido groups using 50 µM tetraacetyl‐N‐azidoacetylmannosamine (AC_4_ManNAz). Experimental results confirmed that this approach successfully modified approximately 95% of cells with azido groups (Figure 3G and Figure S6). Subsequently, OPBP‐1‐PLGA‐DON nanoparticles were anchored to the cell membrane through click chemistry between the modified DBCO on the surface of PLGA nanoparticles and the azido groups on T cells. As shown in the confocal results in Figure 3H and the flow cytometry results in Figure 3I, the nanoparticles were stably anchored to the cell surface through this method, verifying the successful construction of OPBP‐1‐PLGA‐DON‐T cells. Scanning electron microscope (SEM) examination estimated that 110–150 nanoparticles are loaded onto a single T cell (Figure 3J), assuring effective nanodrug anchoring and T cell modification.

### 2.3 Enhanced Functionality of OPBP‐1‐PLGA‐DON‐T Cells

**Figure 4:**
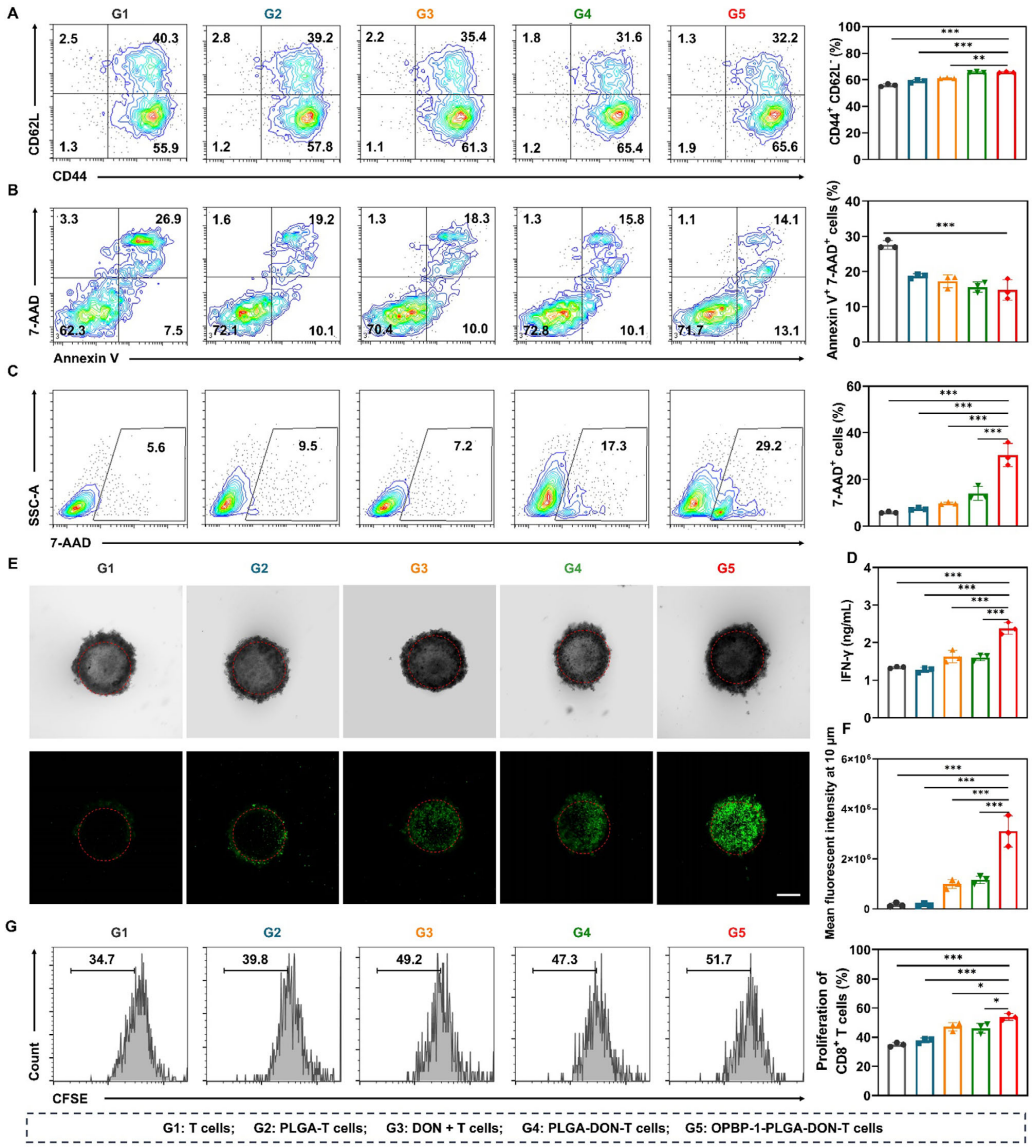
In vitro functional validation of OPBP‐1‐PLGA‐DON‐T cells. (A) T cell memory differentiation was analyzed by flow cytometry using CD44 and CD62L markers after 72 h of treatment. (B) Apoptosis of T cells were analyzed using Annexin V and 7‐AAD staining via flow cytometry after 5 days of treatment. (C) Killing efficiency of T cells against B16‐OVA cells was determined by identifying dead tumor cells using 7‐AAD staining analyzed via flow cytometry. (D) IFN‐γ secretion quantified by ELISA after 48 h of co‐culture with tumor cells. (E) CFSE‐stained CD8^+^ T cells from Groups G1 (T cells), G2 (T cells with PLGA nanoparticles), G3 (T cells with free DON), G4 (T cells with DON‐loaded PLGA), and G5 (T cells with OPBP‐1‐PLGA‐DON) were co‐cultured with B16‐OVA tumor spheroids. Confocal microscopy visualized T‐cell infiltration (green, CFSE‐stained; scale bar: 200 µm). (F) Quantitative analysis of green fluorescence intensity within tumor spheroids. All the results shown are compared with the control group. (G) T‐cell proliferation analyzed by flow cytometry after co‐culture with antigen‐presenting dendritic cells. Dendritic cells (DCs) pre‐treated with antigens were co‐cultured with CFSE‐labeled T cells under various conditions for 48 h, and changes in CFSE fluorescence of the T cells were analyzed by flow cytometry.Green indicates CFSE‐stained CD8^+^ T cells. Scale bar: 200 µm. n = 3, Statistical significance was calculated using a one‐way analysis of variance (ANOVA) followed by Tukey's post hoc test for multiple comparisons. ^*^ *P* < 0.05, ^**^ *P* < 0.01, ^***^ *P* < 0.001.

To robustly investigate the impact of constructing T cell‐drug conjugates, we established five experimental groups (**Figure** **4**). T cells covalently conjugated with DON‐loaded PLGA nanoparticles (**G4**) demonstrated superior effects on CD44^+^CD62L^−^ memory effector T cell (TEM) differentiation (Figure 4A) and exhibited a marked reduction in apoptosis (Figure 4B). Additionally, T cell‐mediated tumor cell killing was significantly enhanced in **G4**, as shown in Figure 4C, with 7‐AAD staining indicating a higher percentage of B16‐OVA tumor cell death. This was further supported by increased IFN‐γ secretion (Figure 4D) compared to DON‐treated T cells in the medium (**G3**), highlighting the enhanced modulatory efficiency provided by nanodrug conjugation on T cells through localized DON release.

Remarkably, anchoring PLGA nanoparticles alone on T cells (**G2**) significantly enhanced T cell function and proliferation in various aspects, suggesting an additional, unidentified mechanism at play. Incorporating DON into PLGA nanocarriers (**G4**) further amplified these beneficial effects. The modification with the PD‐L1 blocking peptide OPBP‐1 (**G5**) extended these benefits even further, showcasing a potent synergy between DON and OPBP‐1 for a more robust anti‐tumor effect.

We also evaluated the infiltration capability of OPBP‐1‐PLGA‐DON‐T cells using 3D tumor spheroids. Tumor spheroids were generated by centrifugation to achieve a compact structure. CFSE‐labeled T cells were co‐cultured with these spheroids, and their infiltration was assessed using confocal microscopy by examining the distribution of green fluorescence within the spheroids. The results indicated that these T cells penetrated deeper into tumor spheroids (Figures 4E,F) with the combined aid of both DON and OPBP‐1. While DON alone promoted T cell migration and adhesion, the modification with OPBP‐1 further enhanced these processes by PD‐1/PD‐L1 interactions, significantly improving T cell penetration into the 3D tumor spheroids.

Furthermore, OPBP‐1 liberated T cells from PD‐1/PD‐L1 interactions with dendritic cells (DCs), enhancing DC antigen cross‐presentation and stimulating T‐cell proliferation (Figure 4G). All these findings underscore the transformative potential of T cell‐drug conjugates in improving ACT outcomes, offering a powerful approach against solid tumors.

### 2.4 Enhanced Tumor Targeting of OPBP‐1‐PLGA‐DON‐T Cells

Due to their nanostructure and physicochemical properties, nanoparticles are easily cleared by the immune phagocytic system of macrophages, neutrophils, and other effector cells in the body.^[^ ^17^ ^]^ Approximately 40% of particles around 200 nm in size are cleared within three days after entering the body.^[^ ^18^ ^]^ We hypothesize that attaching nanodrugs to T cells could circumvent this issue.

**Figure 5:**
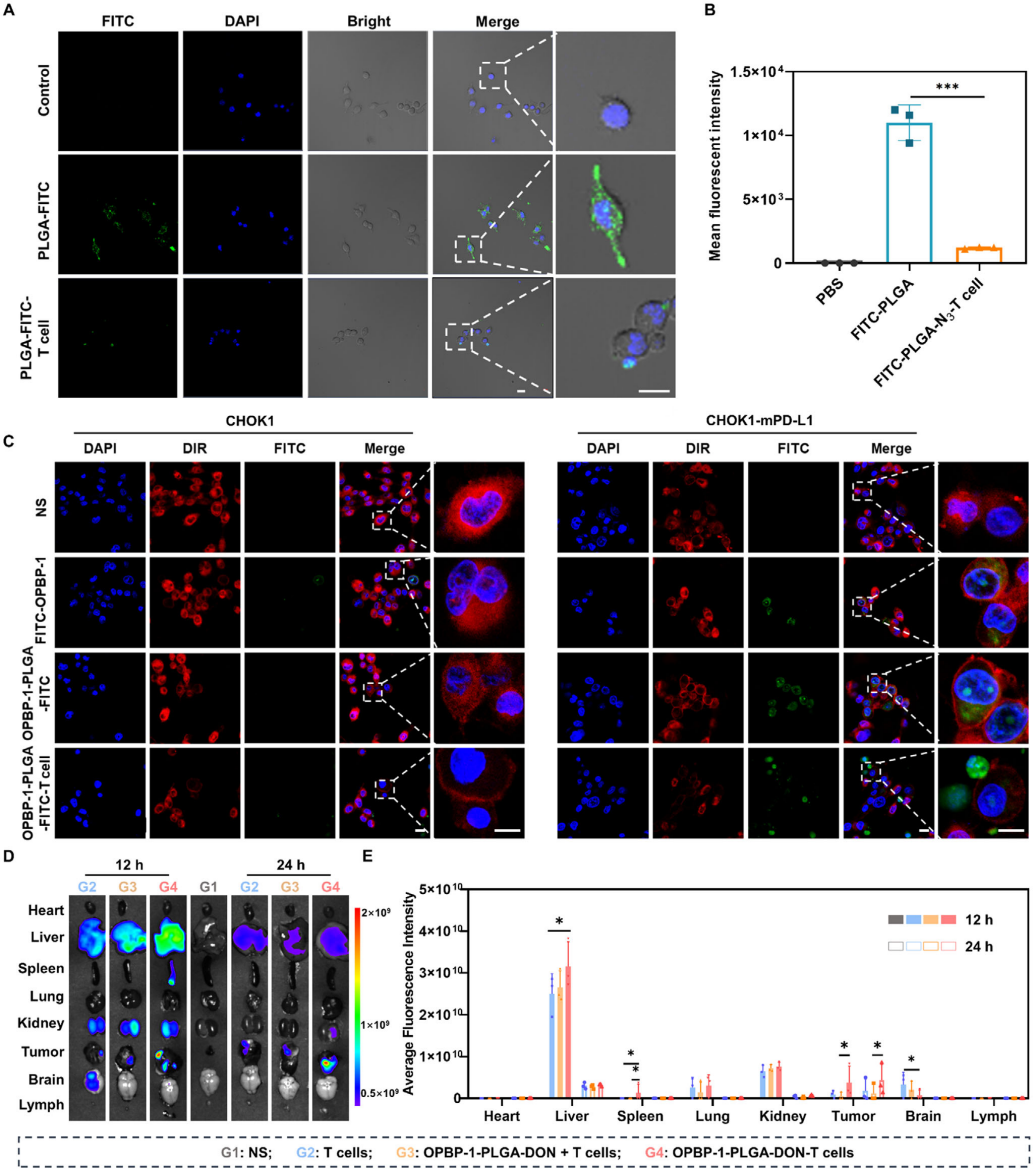
Targeting and in vivo distribution of OPBP‐1‐PLGA‐DON‐T cells. (A) Phagocytosis of free or T‐cell‐carried PLGA‐FITC nanoparticles (green) by RAW264.7 macrophage cell line. Scale bar: 20 µm. (B) Quantification of intracellular FITC fluorescence in RAW264.7 cells. (C) FITC‐labeled OPBP‐1, OPBP‐1‐PLGA‐FITC, and OPBP‐1‐PLGA‐FITC‐T cells were co‐incubated with CHOK1 cells (left) and PD‐L1 overexpressing CHOK1‐mPD‐L1 cells (right) for 30 min, followed by confocal imaging. The cell membrane is shown in red, the nucleus in blue, and OPBP‐1, OPBP‐1‐PLGA‐FITC, and OPBP‐1‐PLGA‐FITC‐T cell in green. Scale bar: 30 µm. (D) In vivo distribution of NS (G1), T cell alone (G2), T cells with free OPBP‐1‐PLGA‐DON nanodrugs (G3) and OPBP‐1‐PLGA‐DON conjugated T cells (G4) in B16‐OVA tumor‐bearing mice after intravenous administration for 12 h and 24 h. T cells were modified with DBCO‐Cy5 for fluorescence evaluation. (E) Quantification of Cy5 fluorescence intensity in various organ tissues. n = 3, Statistical significance was calculated using a one‐way ANOVA followed by Tukey's post hoc test for multiple comparisons. ^*^ *P* < 0.05, ^***^ *P* < 0.001.

To test this, we loaded FITC into PLGA nanoparticles (PLGA‐FITC) and examined the phagocytosis of fluorescent nanoparticles by macrophages under different conditions in vitro. The results demonstrated a significant reduction in the phagocytosis of nanoparticles when attached to T cells compared to freely dispersed nanoparticles (**Figures** **5**A,B). This suggests that the T‐cell conjugation strategy can markedly improve the in vivo circulation and therapeutic efficacy of nanodrugs compared to conventional nanoparticle administration.

We further refined this approach by modifying the surface of the nanoparticles with the PD‐L1 blocking peptide OPBP‐1 and tested its co‐localization using affinity experiments with CHOK1 and CHOK1‐mPD‐L1 cells (PD‐L1 overexpressing cells). The results showed that both OPBP‐1 alone and OPBP‐1‐PLGA‐FITC co‐localized with CHOK1‐mPD‐L1 cells. Furthermore, T cells conjugated with OPBP‐1‐PLGA‐FITC were significantly enriched around PD‐L1 overexpressing cells (Figure 5C), confirming the enhanced targeting of T cells to tumors.

We investigated the in vivo distribution of OPBP‐1‐PLGA‐DON‐T cells following transfusion, labeling the T cells with Cy5 fluorescence via bioorthogonal reactions. The fluorescence intensity in various organs was analyzed at 12 and 24 h post‐reinfusion to assess T cell distribution. At 12 h, higher fluorescence intensity was observed in the liver and kidneys for all groups, likely reflecting the initial biodistribution of infused cells to major filtering and circulation organs. Notably, the OPBP‐1‐PLGA‐DON‐T cell group (**G4**), where OPBP‐1‐PLGA‐DON nanodrugs were covalently conjugated to T cells, exhibited significantly higher fluorescence intensity within the tumor compared to the T cell alone group (**G2**) and the group with free OPBP‐1‐PLGA‐DON nanodrugs (**G3**). This indicates that T‐cell‐nanodrug conjugation enhanced T‐cell accumulation in tumor tissues (Figure 5D,E). By 24 h, fluorescence intensity in non‐tumor organs, such as the liver and kidneys, decreased, whereas tumor retention for the conjugate group (**G4**) remained substantial and consistent (Figure 5E). This enhanced tumor enrichment underscores the dual advantage of the conjugate system: it not only reduces the phagocytic clearance of nanodrugs by the immune system but also improves the targeting of both T cells and nanodrugs to tumors. The mutualistic relationship between T cells and nanodrugs enables each to enhance the other's effectiveness, creating a collaborative and potent therapeutic platform.

### 2.5 Enhanced Antitumor Effects of OPBP‐1‐PLGA‐DON‐T Cells In Vivo

**Figure 6:**
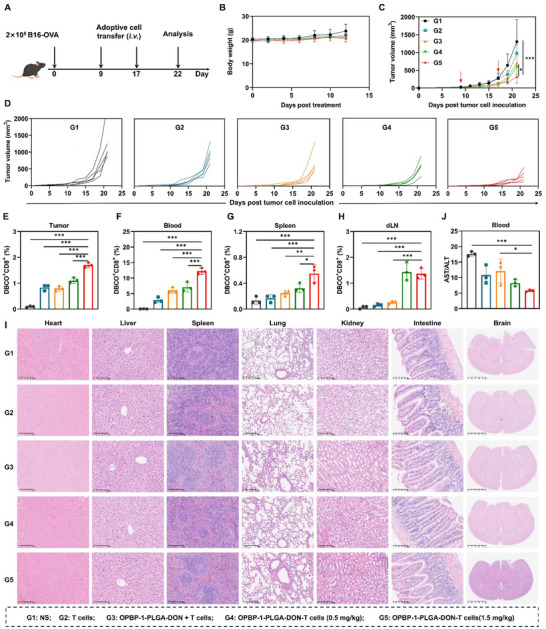
In vivo anti‐tumor activity of OPBP‐1‐PLGA‐DON‐T Cells. (A) Schematic representation of administration with C57BL/6J mice inoculated with B16‐OVA cells. On day 9, mice were randomly divided into 5 groups and underwent ACT on day 9 and 17. Body weight (B) and tumor growth curve (C) after ACT, n = 5. (D) Tumor growth curves for each group of mice, n = 5. Proportion of DBCO^+^ CD8^+^ T cells in tumor (E), blood (F), spleen (G) and lymph node (H) after ACT, n = 3. Data for T‐cell analysis were limited to three mice per group due to insufficient cell numbers from smaller tumors in some treatment groups. (I) HE staining of the heart, liver, spleen, lung, kidney, and small intestine after administration. Scale bar: 200 µm. (J) Ratio of AST/ALT after treatment, n = 3. ALT/AST analysis was performed on the same three mice used for T cell analysis to maintain consistency. statistical significance was calculated using a one‐way ANOVA followed by Tukey's post hoc test for multiple comparisons. ^*^ *P* < 0.05, ^**^ *P* < 0.01, ^***^ *P* < 0.001.

To elucidate the antitumor efficacy of OPBP‐1‐PLGA‐DON‐T cells following T‐cell reinfusion, we established a subcutaneous melanoma B16‐OVA tumor model in C57BL/6J mice (**Figure** **6**A). The experimental cohorts included normal saline (**G1**), CD8^+^ T cells derived from OT‐1 mice (**G2**), free OPBP‐1‐PLGA‐DON and T cells (**G3**), and OPBP‐1‐PLGA‐DON‐T cells at dose of 0.5 mg k^−1^g (**G4**) and 1.5 mg k^−1^g of DON (**G5**), administered twice for ACT. Throughout the treatment regimen, the body weights of the mice remained stable (Figure 6B). Tumor volume analysis revealed that the T‐cell‐nanodrug conjugates (**G4** and **G5**) markedly inhibited tumor growth compared to pure T cells (**G2**, Figures 6C‐D and S7). We conducted a survival study using the B16‐OVA tumor model (Figure S8), where the conjugate groups with 0.5 mg k^−1^g and 1.5 mg k^−1^g DON exhibited significantly improved survival rates compared to T cells alone and T cells with free nanodrugs, highlighting their enhanced therapeutic potential.

Furthermore, the quantity of infused T cells labeled with Cy5‐DBCO in tumors, blood, spleen, and lymph nodes was significantly higher in all conjugate groups (**G4** and **G5**) than in the non‐conjugate group (**G3**, Figures 6E‐H). This suggests that sustained glutamine inhibition enhances T cell persistence and infiltration into solid tumors by promoting migration and altering T cell metabolism.

Given that DON nanodrug conjugates are anchored to T cells for enhanced modulation, we were able to use a lower dose of DON compared to other studies.^[^ ^19^ ^]^ Safety assessments, including HE staining of various tissues and liver function tests (Figures 6I,J), demonstrated that this reduced‐dose T‐cell‐nanodrug conjugate system did not induce organ damage and maintained normal physiological levels of AST and ALT post‐treatment.

In clinical contexts, DON's off‐target effects have limited its use due to adverse reactions. Our findings indicate that the T‐cell‐nanodrug conjugate approach significantly improves DON targeting to tumor tissues via OPBP‐1‐mediated binding to PD‐L1 and TCR‐mediated binding to the OVA antigen on tumor cells. Consequently, our system achieves targeted, temporal, and spatial regulation of T‐cell glutamine metabolism, significantly amplifying its antitumor effects while enabling the precise delivery of DON for synergistic tumor eradication, thereby mitigating its systemic toxic effects.

### 2.6 Enhanced Systemic Immune Response and Antitumor Efficacy of OPBP‐1‐PLGA‐DON‐T Cells

**Figure 7:**
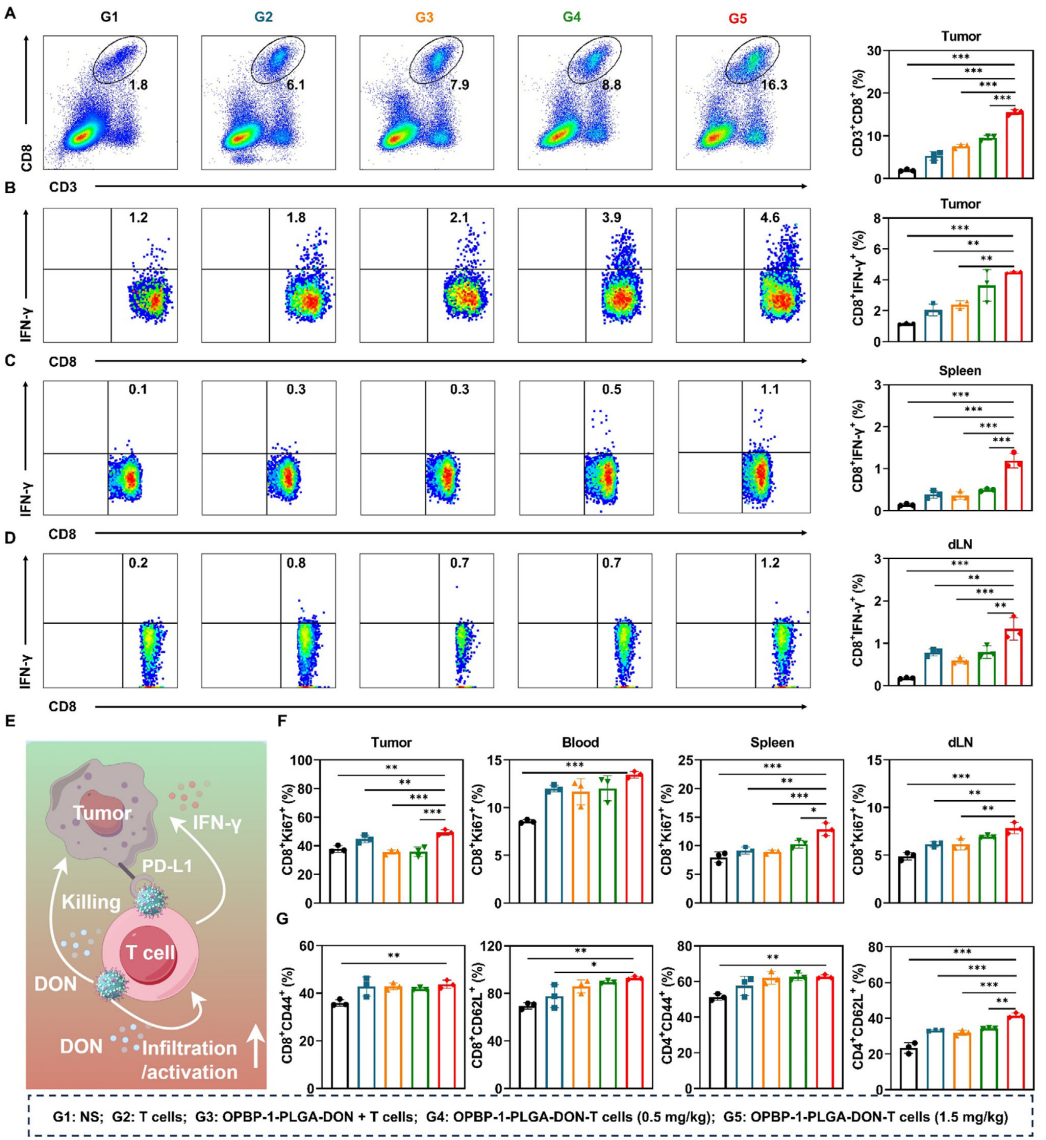
Enhanced systemic immune response and antitumor efficacy of OPBP‐1‐PLGA‐DON‐T Cells. (A) Proportion of CD8^+^ T cells within tumor tissue determined by flow cytometry after incubating tumor single‐cell suspensions with fluorescent antibodies. Proportion of CD8^+^ IFN‐γ^+^ T cells isolated from tumor tissue (B), spleen (C) and draining lymph node (D) of tumor‐bearing mice after stimulation and activation. (E) Schematic representation of the mechanism by which OPBP‐1‐PLGA‐DON conjugation enhances antitumor effects of T cells. The figure was generated using Figdraw. (F) Expression of Ki67 in T cells isolated from various tissues after administration. (G) The proportion of CD44 and CD62L, which represent memory‐like cell expression in T cells isolated from the spleen determined by flow cytometry. n = 3, statistical significance was calculated using a one‐way ANOVA followed by Tukey's post hoc test for multiple comparisons. ^*^ *P* < 0.05, ^**^ *P* < 0.01, ^***^ *P* < 0.001.

We also assessed the systemic effects of OPBP‐1‐PLGA‐DON‐T cells on overall CD8^+^ T cell function and antitumor activity. Treatment with OPBP‐1‐PLGA‐DON‐T cells significantly increased the total CD8^+^ T cell population within tumors, with a dose‐dependent enhancement observed as DON concentration increased (**Figure** **7**A).

Intracellular cytokine staining revealed that OPBP‐1‐PLGA‐DON‐T cells significantly boosted the secretion of IFN‐γ by tumor‐infiltrating CD8^+^ T cells (Figure 7B). Although the overall percentage of IFN‐γ positive cells in tumors was low due to the limitations of the *ex vivo* stimulation method,^[^ ^20^ ^]^ which involved 1 µg/mL OVA peptide for 6 h, the conjugated group (**G5**) still showed a significant improvement. IFN‐γ positive cells increased approximately fourfold compared to the NS group (**G2**) and doubled compared to the free nanodrug and T cell group (**G3**). Additionally, this increase in IFN‐γ secretion was also observed in CD8^+^ T cells within the spleen (Figure 7C) and dLN (Figure 7D), thereby enhancing the proportion of activated T cells across these key immune sites. These findings suggest that the transferred T cells are not only effective locally within the tumor microenvironment but also contribute to a systemic antitumor response, amplifying the overall immune attack on the cancer.

Given the potential for T cell exhaustion following reinfusion, we further evaluated the proliferation and memory‐like characteristics of T cells across various tissues. The results demonstrated that OPBP‐1‐PLGA‐DON‐equipped CD8^+^ T cells exhibited enhanced proliferation (Figure 7E‐F), while both CD8^+^ and CD4^+^ T cells exhibited significantly enhanced memory‐like features (Figure 7G). These enhanced properties are crucial for sustaining long‐term antitumor immunity and preventing relapse.

### 2.7 Antitumor Efficacy of OPBP‐1‐PLGA‐DON‐T Cells in Colorectal Cancer and Lymphoma Tumor Models

**Figure 8:**
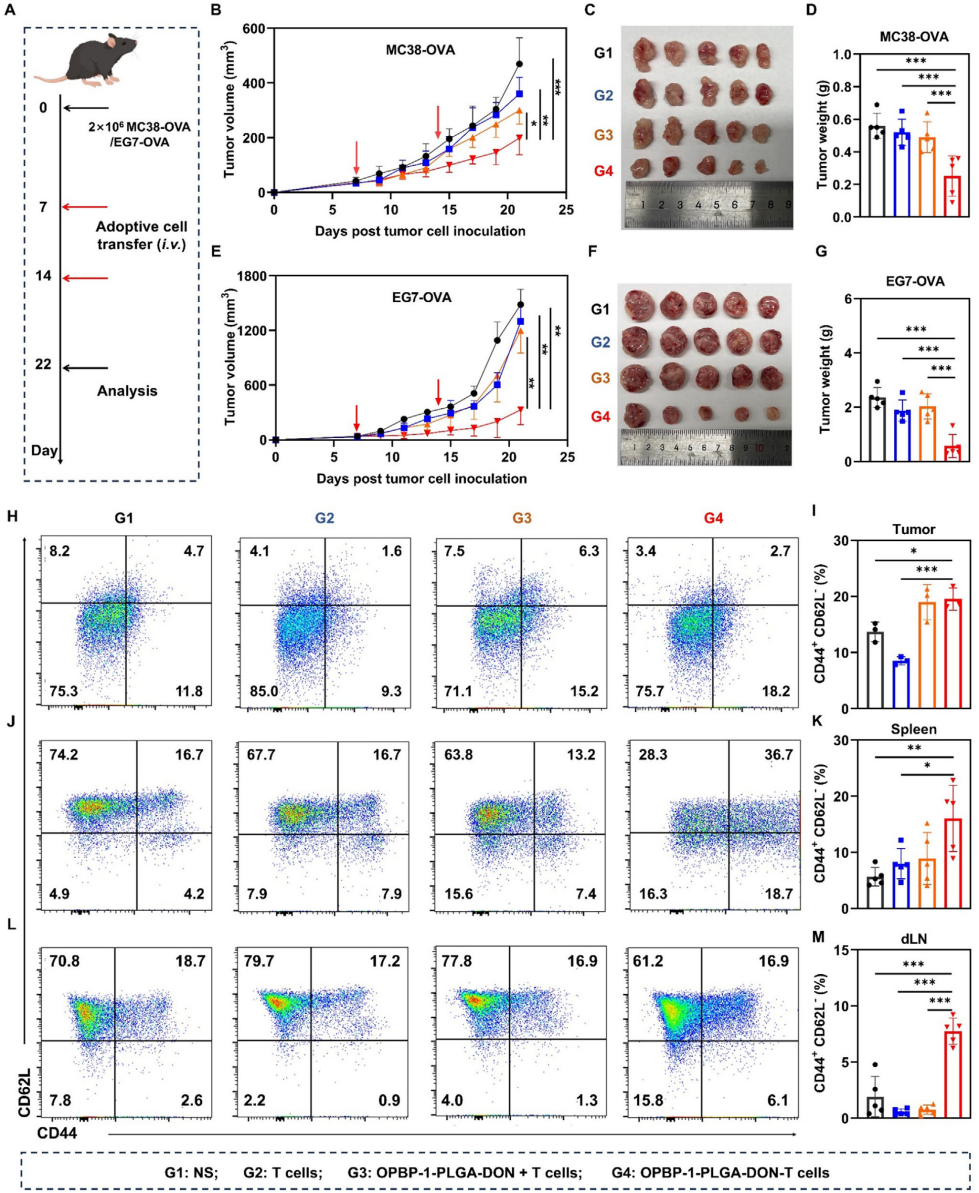
Antitumor efficacy of OPBP‐1‐PLGA‐DON‐T Cells in colorectal cancer and lymphoma models. (A) Schematic of the administration protocol: C57BL/6J mice were inoculated with MC38‐OVA or EG7‐OVA cells on the right dorsal side. After 7 days, mice were randomly divided into four groups, and adoptive transfer therapy was performed on days 7 and 14. (B) Tumor growth curve for MC38‐OVA tumor‐bearing mice. (C) Representative images of MC38‐OVA tumor size following adoptive T cell therapy. (D) Statistical analysis of tumor size shown in panel (C). (E) Tumor growth curve for EG7‐OVA tumor‐bearing mice. (F) Representative images of EG7‐OVA tumor size following adoptive T cell therapy. (G) Statistical analysis of tumor weight shown in panel (F). Flow cytometry analysis of CD44 and CD62L expression on CD8^+^ T cell subsets within the tumor (H), the spleen (J) and the DLN (L). Data for T‐cell analysis (H) were limited to three mice per group due to insufficient cell numbers from smaller tumors in some treatment groups. Statistical analysis of the proportion of TEM (CD44^+^ CD62L^−^) among CD8^+^ T cells in the tumor (I), the spleen (K) and the DLN (M). n = 5 for all panels, with statistical significance determined using a one‐way ANOVA followed by Tukey's post hoc test for multiple comparisons. ^*^ *P* < 0.05, ^**^ *P* < 0.01, ^***^ *P* < 0.001.

To comprehensively evaluate the therapeutic potential of OPBP‐1‐PLGA‐DON‐T cells, subcutaneous colorectal cancer (MC38‐OVA) and lymphoma (EG7‐OVA) tumor models were established in C57BL/6J mice (**Figure** **8**A). Following two infusions, this treatment did not affect the body weight of the mice (Figure S9), indicating good safety. Importantly, the OPBP‐1‐PLGA‐DON‐T cell conjugate group exhibited significantly inhibited tumor growth compared to the control groups in both colorectal cancer‐bearing mice (Figures 8B‐D) and lymphoma‐bearing mice (Figures 8E‐G).

Previously, we demonstrated that both free DON and the conjugation of OPBP‐1‐PLGA‐DON on T cells promote the differentiation of T cells into TEM. Further analysis within the tumor microenvironment (Figure 8H‐I) revealed that the proportion of TEM in the OPBP‐1‐PLGA‐DON‐T cells (**G4**) was slightly higher than in T cells with free nanodrugs (**G3**), but this difference was not statistically significant, indicating that both treatments similarly enhance T cell memory differentiation within tumors. In contrast, in the spleen (Figure 8J,K) and lymph nodes (Figure 8L,M), the OPBP‐1‐PLGA‐DON‐T cells (**G4**) exhibited a significantly higher proportion of TEM compared to T cells with free nanodrugs (**G3**). Specifically, in the spleen, TEM levels were 16.06 ± 4.93% in the **G4** group compared to 8.92 ± 3.72% in the **G3** group, and in the lymph nodes, TEM levels reached 7.75 ± 0.92% in the **G4** group compared to 0.76 ± 0.32% in the **G3** group. A similar trend was observed among CD4^+^ T cells, with significantly elevated TEM proportions in the spleen (Figures S10A,B) and lymph nodes (Figures S10C,D). The increased presence of TEM across multiple tissues demonstrates that OPBP‐1‐PLGA‐DON conjugation effectively promotes T cell differentiation into memory phenotypes. This adoptive transfer therapy with T cell‐nanodrug conjugates enhances the generation of immune memory cell populations, leading to a more robust and sustained anti‐tumor response.

## 3 Conclusion

We have developed a novel T‐Cell‐PD‐L1‐DON‐nanodrug conjugate system that significantly enhances T‐cell functionality and anti‐tumor efficacy. This innovative approach enables precise in situ regulation of T‐cell metabolism, promoting differentiation into memory phenotypes and improving tumor infiltration. The incorporation of PD‐L1 blocking peptides on the surface alleviates immune suppression, amplifying tumor‐targeting capabilities and boosting overall anti‐tumor responses. By dual‐targeting tumors through the TCR and OPBP‐1, these conjugates effectively concentrate DON within tumors, synergistically enhancing T‐cell‐mediated tumor cell elimination while minimizing off‐target effects.

Crucially, our nanodrug conjugates offer a comprehensive solution by modulating T‐cell phenotype, proliferation, function, motility, and infiltration, tackling the formidable challenges of ACT in solid tumor treatment with a single, cohesive strategy. Given the established clinical use of PLGA nanoparticles, our design holds the potential for future clinical translation.

This T Cell‐nanodrug conjugate system represents a versatile and powerful platform, capable of integrating diverse therapeutic agents for highly personalized treatments tailored to the unique molecular and immunological landscapes of individual tumors. Future research will be pivotal in validating its efficacy across various tumor types and in exploring synergistic combinations with other therapies, such as innate immunity activators, to unlock even greater therapeutic potential.

## 4 Experimental Section

### Cell Lines and Mice

MC38‐OVA cells were kindly provided by Prof. Changzheng Lu (Shenzhen Bay Laboratory, China). EG7‐OVA cells were kindly provided by Prof. Shengdian Wang (Institute of Biophysics, Chinese Academy of Sciences, China). Cell line stably expressed human PD‐1 (CHO‐K1‐mPD‐L1) on the cell membrane was established from CHO‐K1 cells transfected with lentiviral vector pLVX‐Puro. B16‐OVA cells and CHOK1 cells were cultured in RPMI 1640 (Gibco, Grand Island, USA) consisting of 10% fetal bovine serum (Sigma, USA), 100 µg/mL streptomycin (Solarbio, China) and 100 U/mL penicillin (Solarbio, China), in an incubator with 98% humidity and 5% CO_2_ at 37 °C.

C57BL/6J mice were maintained in the specific pathogen‐free facility at Sun Yat‐sen University. OT‐1 mice (OVA_257–264_ TCR transgenic mice) were kindly provided by Prof. Xuanming Yang (Shanghai Jiao Tong University, China). Animal experimental procedures were carried out following the national and institutional guidelines and were approved by the Ethics Committee of Sun Yat‐sen University (SYSU‐YXYSZ20230605).

### Cytotoxicity Assays

The cytotoxicity of DON (MedChemExpress, USA) on B16‐OVA cells was assessed using a standard MTT assay. Initially, freshly trypsinized cells were seeded into flat‐bottom 96‐well plates at a density of 1.5 × 10^4^ cells per well. Subsequently, varying concentrations of DON ranging from 0.3 µM to 100 µM were added to the wells, followed by incubation at 37 °C for 24, 48, and 72 h. After the respective incubation periods, 20 µL of MTT solution was added to each well, and the plates were further incubated at 37 °C for an additional 4 h. Following this incubation, the supernatant was carefully removed, and 150 µL of dimethyl sulfoxide (DMSO) was added to dissolve the purple formazan crystals formed by viable cells. The plates were then shaken in the dark for 15–20 mins to ensure complete dissolution of the crystals. The absorbance was measured at 490 nm using the microplate photometer (Thermo Scientific, USA) within 15 min.

The cytotoxicity of DON on T cells was evaluated using a Cell Counting Kit‐8 (CCK‐8) assay. Initially, T cells were isolated from the spleen and lymph nodes of mice and prepared as a single‐cell suspension at a concentration of 5 × 10^4^ cells per well. Subsequently, 200 µL of the cell suspension was evenly distributed into flat‐bottom 96‐well plates, and varying concentrations of DON ranging from 0.3 µM to 100 µM were added to the wells. The plates were then incubated at 37 °C for 24, 48, and 72 h to allow for exposure to DON. Following the respective incubation periods, 20 µL of CCK‐8 solution was added to each well and the plates were further incubated at 37 °C for 2 h to allow for cell staining. After incubation with CCK‐8, the absorbance of each well was measured at 450 nm using the microplate photometer (Thermo Scientific, USA) within 15 min.

### T Cell Differentiation and Apoptosis

T cells isolated from mouse spleen and lymph node were prepared as a single‐cell suspension at a concentration of 1 × 10^6^ cells. Then, 200 µL of cell suspension was evenly seeded in round‐bottom 96‐well (SORFA, China) plates and supplemented with 100 U/mL of IL‐2 (Peprotech, USA), 1 µg/mL of CD3 (145‐2C11, Biolegend, USA) and CD28 (37.51, Biolegend, USA) antibodies, and 0.3 µM DON. The plates were then incubated at 37 °C for 72 h.^[^ ^21^ ^]^ After the incubation period, cells were collected and stained with anti‐CD8α‐PE (53‐6.7, eBioscience, USA), anti‐CD44‐APC (IIM7, eBioscience, USA), and anti‐CD62L‐Cy5.5 (L‐selectin, eBioscience, USA) fluorescent antibodies for flow cytometry analysis to determine the ratio of T cell effector memory (TEM) to central memory (TCM) cells.

For assessing T‐cell apoptosis, 200 µL of the cell suspension was distributed into round‐bottom wells of a 96‐well plate. 100 U/mL of IL‐2 (Peprotech, USA), 1 µg/mL of CD3 (145‐2C11, Biolegend, USA) and CD28 (37.51, Biolegend, USA) antibodies, and 0.3 µM DON were added to the wells. The cells were then co‐incubated at 37 °C for 5 days in a cell culture incubator. After the co‐incubation period, the cells were harvested, resuspended in 1 × Binding buffer, and stained with 7‐AAD and Annexin V dyes. The stained cells were then incubated at room temperature in the dark for 15 min and subjected to flow cytometry within 1 h.

### T Cell Infiltration and Migration

B16‐OVA cells were added to a 96‐well plate pre‐treated with agarose at a concentration of 8000 cells per well in 100 µL of medium. The plate was centrifuged at 2000 g for 10 min to aggregate the cells into spheres, which were then cultured at 37 °C for 5 days to form tightly packed 3D cell spheres.^[^ ^22^ ^]^ On the fifth day, 0.3 µM DON were added to the culture medium and incubated for 24 h. Then, T cells were stained with CFSE and added to each well at a concentration of 1 × 10^6^ cells per well, followed by 48 h of incubation for cell infiltration. The tumor spheres were then washed twice with PBS and subjected to confocal microscopy for imaging. In a separate set of experiments, T cells stained with CFSE were added directly to the tumor spheres after they formed, followed by 24 h of incubation with 0.3 µM DON. After 48 h of coincubation, the tumor spheres were collected and subjected to confocal microscopy for imaging.

For assessing T‐cell migration, T cells isolated from mouse spleen and lymph nodes were stimulated with 100 U/mL IL‐2 and 1 µg/mL of CD3 and CD28 antibodies for three days. Following stimulation, 200 µL of cell suspension containing 1 × 10^6^ cells was added to the upper chamber of a 6.5 mm transwell with a 5.0 µm pore polycarbonate membrane insert. In the lower chamber, 400 µL of culture medium containing DON or the supernatant from B16‐OVA cells treated with DON for 24 h was added. The transwell plates were then incubated at 37 °C for 48 h to allow for cell migration. After incubation, cells were collected and the number of CD8^+^ T cells was quantified using flow cytometry.^[^ ^23^ ^]^

### RNA Isolation and Library Preparation

Total RNA was extracted using the TRIzol reagent (Invitrogen, CA, USA) according to the manufacturer's protocol. RNA purity and quantification were evaluated using the NanoDrop 2000 spectrophotometer (Thermo Scientific, USA). RNA integrity was assessed using the Agilent 2100 Bioanalyzer (Agilent Technologies, Santa Clara, CA, USA). Then the libraries were constructed using the VAHTS Universal V6 RNA‐seq Library Prep Kit according to the manufacturer's instructions. The transcriptome sequencing and analysis were conducted by OE Biotech Co., Ltd. (Shanghai, China).

### RNA Sequencing and Analysis

The libraries were sequenced on an Illumina Novaseq 6000 platform and 150 bp paired‐end reads were generated. About 55 raw reads for each sample were generated. Raw reads in fastq format were first processed using fastp, and the low‐quality reads were removed to obtain clean reads. Then, about 49 clean reads for each sample were retained for subsequent analyses. The clean reads were mapped to the reference genome using HISAT2. The FPKM of each gene was calculated, and the read counts of each gene were obtained by HTSeq‐count. PCA analyses were performed using R (v 3.2.0) to evaluate the biological duplication of samples.

Differential expression analysis was performed using DESeq2. Q value < 0.05 and foldchange > 2 or foldchange < 0.5 were set as the thresholds for significantly differential expression genes (DEGs). Hierarchical cluster analysis of DEGs was performed using R (v 3.2.0) to demonstrate the expression pattern of genes in different groups and samples. The radar map of the top 30 genes was drawn to show the expression of up‐regulated or down‐regulated DEGs using R packet gradar.

Based on the hypergeometric distribution, GO, KEGG pathway, Reactome, and Wiki Pathways enrichment analyses of DEGs were performed to screen the significant enriched term using R (v 3.2.0), respectively. R (v 3.2.0) was used to draw the column diagram, the chord diagram, and the bubble diagram of the significant enrichment term.

Gene Set Enrichment Analysis (GSEA) was performed using GSEA software. The analysis used a predefined gene set, and the genes were ranked according to the degree of differential expression in the two types of samples. Then it is tested whether the predefined gene set was enriched at the top or bottom of the ranking list.

### Preparation and Characterization of OPBP‐1‐PLGA‐DON Nanodrugs

PLGA (50:50 lactic acid: glycolic acid ratio, MW 7500–17000 Da, Sigma‐Aldrich, USA) nanoparticles were prepared using ultrasonic emulsification, with DON loaded into their structure. The DON content in the supernatant of the nanoparticle preparation using HPLC and calculated the drug loading and encapsulation efficiency according to the following formulas based on the total amounts of DON and PLGA added: $Loading\;ratio=\frac{Weight\;of\;loaded\;DON}{Total\;weight\;of\;PLGA\;and\;DON}$; $Encapsulat\;ion\;efficiency=\frac{Weight\;of\;loaded\;DON}{Weight\;of\;initially\;added\;DON}$. Carboxyl groups were introduced onto the surface of PLGA nanoparticles via 30 min EDC/NHS activation in MES buffer (pH 6.0), followed by three centrifugation washes (15000 rpm, 5 min). The PD‐L1 antagonistic peptide OPBP‐1 and DBCO‐PEG‐NH₂ were sequentially conjugated to the carboxyl groups of PLGA nanoparticles through their amino groups, with each incubation lasting 2 h at 25 °C. Post‐reaction, the nanoparticles were washed three times with PBS (15000 rpm, 5 min) to remove unreacted components. During this process, supernatants were collected to quantify DON content and assess drug loss during surface modification. This conjugation allowed for the anchoring of the DBCO group and the PD‐L1 peptide OPBP‐1 onto the surface of the nanoparticles. The particle size, zeta potential, morphology, and stability of the PLGA nanoparticles were analyzed using techniques including Malvern zeta sizer (ZS90, Malvern Analytical, Malvern, UK) and transmission electron microscopy (H‐7650, Hitachi, Japan). High‐performance liquid chromatography and UV spectrophotometry were employed to investigate the encapsulation and loading efficiency of the drug by PLGA nanoparticles, enabling the optimization of preparation methods to obtain ideal OPBP‐1‐PLGA‐DON nanodrugs. One mL of OPBP‐1‐PLGA‐DON and an equivalent concentration of DON aqueous solution were added into a dialysis bag (8000‐14000 M), and placed in 100 mL of PBS at 37 °C and kept gently stirring to simulate drug release. At 0.5 h, 1 h, 2 h, 4 h, 6 h, 8 h, 10 h, 12 h, 1d, 2 d, 3 d, 4 d, 5 d, 6 d, 7 d, 8 d, 9 d and 10 d, 1 mL of release medium samples were withdrawn and replaced with 1 mL of fresh release medium. After filtration, the content of the samples was detected by an RP‐HPLC (Alliance E2695, Waters, USA), and the cumulative drug release of DON in vitro was calculated.^[^ ^24^ ^]^

### Quantification of peptides on PLGA nanoparticles

FCS was performed using a Zeiss LSM 510 microscope (LSM 510‐META/Confocor2, Carl Zeiss, Jena, Germany) with settings adjusted as follows: emission signals for FCS were recorded without the beam splitter, using the appropriate filter sets. The pinholes were optimized to maximize the count rate, using free dye in PBS, with sample volumes typically set to 5 µL. Fluorescent fluctuations over time were recorded for 30 intervals of 10 seconds each. The raw data were processed and analyzed using ConfoCor3 software. Autocorrelation curves were fitted using a two‐component model (equation 3). Diffusion times for free Atto488‐labeled OPBP‐1 and its conjugated PLGA nanoparticles were fixed during the fitting process. The number of OPBP‐1 peptides per PLGA nanoparticle was determined by dividing the molecular brightness of Atto488‐OPBP‐1‐PLGA (in counts per molecule, CPM) by the CPM of freely diffusing Atto488‐OPBP‐1. The fraction of free dye or free Atto488‐OPBP‐1 was below 1% and was excluded from the analysis.

(1) $$\begin{array}{ccc} G_{2comp}\left(\tau \right) & = & 1+\frac{1}{N}\cdot \left(\;1+\frac{T_{trip}}{1-T_{trip}}e^{-\frac{\tau }{\tau_{trip}}}\;\right)\\ & & \cdot \left[\;\frac{f_{1}}{\left(\;1+\frac{\tau }{\tau_{D_{1}}}\;\right){\left(\;1+\frac{\tau }{S^{2}\tau_{D_{1}}}\;\right)}^{1/2}}+\frac{f_{2}}{\left(\;1+\frac{\tau }{\tau_{D_{2}}}\;\right){\left(\;1+\frac{\tau }{S^{2}\tau_{D_{2}}}\;\right)}^{1/2}}\;\right] \end{array}$$

where *G*_2*comp*_(τ) is the two‐component autocorrelation function, *N* is the number of particles, S the structural parameter, *T_trip_* is the fraction of fluorophores in the triplet state, τ_*trip*_ is the corresponding triplet time, f1 and f2 are the fraction of the particles of the corresponding component 1 or 2, $\tau_{D_{1}}$ and $\tau_{D_{2}}$ are the diffusion times of the corresponding component 1 or 2.

### Blocking Activity of OPBP‐1‐PLGA

The PD‐1 overexpressing cells CHO‐K1‐PD‐1 previously established by our group, and the PD‐L1 proteins with Fc tag of IgG1 (Sino Biological, Beijing, China) were used. Peptides or OPBP‐1‐PLGA were diluted to a concentration of 100 µM using normal saline and incubated with 50 ng of PD‐L1‐Fc on ice for 30 minutes. This mixture was then incubated with CHO‐K1‐PD‐1 cells on ice for an additional 30 mins, followed by treatment with phycoerythrin (PE)‐conjugated goat anti‐human IgG1 antibodies (anti‐Fc‐PE) (eBioscience). Cells incubated only with the anti‐Fc‐PE antibody were used as negative controls to account for non‐specific fluorescence. For positive controls, cells were incubated with PD‐1‐Fc and anti‐Fc‐PE in the absence of peptides to represent the strongest PD‐1/PD‐L1 protein binding. The mean fluorescent intensity (MFI) of the cells was measured using a BD FACSLSRForte (BD Biosciences, USA) flow cytometer and used to calculate blocking efficacy. The blocking efficacy (%) was calculated using the formula: the blocking efficacy (%) = (MFI of the positive control – MFI of the tested peptides)/ MFI of the positive control × 100%.

### Preparation and Characterization of OPBP‐1‐PLGA‐DON‐T Cells

T cells isolated from mouse spleen and lymph nodes were co‐incubated with varying concentrations of acetylated N‐azidoacetylgalactosamine (AC_4_ManNAz) for 3 days. DBCO‐Cy5 was then added to assess azido modification efficiency. T cells with different azido densities (N_3_‐T cells) were obtained using the aforementioned method. OPBP‐1‐PLGA‐DON nanoparticles were modified with DBCO through the amide reaction and incubated with N_3_‐T cells at 37 °C for 1 hour to allow the attachment of different quantities of OPBP‐1‐PLGA‐DON nanoparticles to the T cell surface. Thiourea fluorescein was used as a model drug encapsulated in the nanoparticles. The binding efficiency and stability of the nanoparticles on the T cell surface were examined using flow cytometry and laser confocal fluorescence microscopy.

### Phagocytosis by Macrophages

Physiological saline, free PLGA‐FITC nanoparticles, and PLGA‐FITC nanoparticles loaded onto T cells were separately co‐incubated with the macrophage cell line RAW264.7. Following the co‐incubation period, the phagocytosis of both nanoparticles and T cell “backpacks” by RAW264.7 cells was visualized and analyzed using confocal microscopy.

### Functions of OPBP‐1‐PLGA‐DON‐T Cells

The effects of OPBP‐1‐PLGA‐DON conjugates on T cell proliferation, differentiation, function, and infiltration capacity into 3D tumor models were evaluated using CFSE staining, immunophenotypic analysis, flow cytometry, and confocal microscopy.

### In Vivo Distribution T‐cell‐nanodrug conjugates

OPBP‐1‐PLGA nanoparticles were constructed and loaded onto CD8^+^ T cells isolated from OT‐1 mice. The distribution of fluorescently labeled T cell “backpacks” in tumor‐bearing mice was detected using an in vivo imaging system, and dynamic distribution in organs such as tumors, hearts, and livers was evaluated.

### In Vivo Antitumor Efficacy of OPBP‐1‐PLGA‐DON T‐Cells

B16‐OVA cells were cultured and passaged, and single‐cell suspensions were prepared. These cells were then subcutaneously injected into the right dorsal region of C57BL/6J mice aged 5–8 weeks. When tumor volume reached 30–40 mm^3^ and was relatively uniform, mice were grouped for antitumor therapy. Therapy included physiological saline, T cells, OPBP‐1‐PLGA‐DON + T cells, and high and low doses of OPBP‐1‐PLGA‐DON loaded T cells (OPBP‐1‐PLGA‐DON‐T cells), with tail vein injections of 3 × 10^6^ T cells on days 9 and 17. The MC38‐OVA and EG7‐OVA models were established with four treatment groups: saline, T cells, OPBP‐1‐PLGA‐DON + T cells, and OPBP‐1‐PLGA‐DON (1.5 mg k^−1^g) + T cells, with tail vein injections of 3 × 10^6^ T cells on days 7 and 14. Tumor size, mouse weight, survival period, and antitumor effects were recorded. Tumor tissues were dissected, digested, and processed for single‐cell suspensions to evaluate CD8^+^ T cell infiltration using flow cytometry. To determine the intracellular IFN‐γ and Ki67 of CD8^+^ T cells from tumor tissue, lymphocytes were separated by Percoll (GE Healthcare, USA). The isolated lymphocytes were carefully collected, washed twice with PBS (pH 7.2), and plated in a 24‐well plate (1 × 10^6^ cells/well). After adding 1 µL of protein transport inhibitor cocktail (BD, USA), the cells were stimulated by 1µg/mL OVA peptide for 6 h. Cells were then collected and incubated with anti‐CD3‐eFlour710 (17A2, eBioscience, USA), anti‐CD8α‐PE (53‐6.7, eBioscience, USA), anti‐CD44‐APC (IIM7, eBioscience, USA) and anti‐CD62L‐Cy5.5 (L‐selectin, eBioscience, USA). After 30 mins of incubation, cells were washed and adhered for another 30 min at room temperature. Then 800 µL of permeabilization buffer was added to the cells. Anti‐IFN‐γ‐APC (XMG1.2, eBioscience, USA) and anti‐Ki67‐Pc7 (SolA15, eBioscience, USA) were added for intracellular staining on ice for 30 min. The proportion of lymphocytes in the tumor tissue was measured by flow cytometry after being washed twice with PBS (pH 7.2). We first gated on CD3^+^CD8^+^ cells and then calculated the proportion of CD44^+^CD62L^−^ or IFN‐γ^+^ cells, comparing this with the isotype control to identify the target cell population.

Cells from lymph nodes or spleens (2 × 10^6^ cells/well) were cultured with RPMI‐1640 (Gibco, Grand Island, USA) containing 10% FBS. As described above, cells were treated by a protein transport inhibitor cocktail and stimulated by 1µg/mL OVA peptide for 6 h. Cells were then collected and incubated with anti‐CD3‐eFlour710 (17A2, eBioscience, USA) and anti‐CD8α‐PE (53‐6.7, eBioscience, USA). After 30 mins of incubation, cells were washed and fixed for another 30 min at room temperature. Then 800 µL of permeabilization buffer was added to the cells. Anti‐IFN‐γ‐APC (XMG1.2, eBioscience, USA) and anti‐Ki67‐Pc7 (SolA15, eBioscience, USA) were added for intracellular staining on ice for 30 min. The proportion was measured by flow cytometry (Cytoflex, Beckman Coulter, USA) after being washed twice with PBS (pH 7.2).

### Statistical analysis

Differences between the two groups were statistically analyzed using Graphpad 8.0.2 software with one‐sided Mann–Whitney test. Comparisons of more than two groups were done with one‐way ANOVA analysis followed by Tukey's multiple comparisons test. All data were shown as means ± SD. ^*^ *P* < 0.05, ^**^ *P* < 0.01, and ^***^ *P* < 0.001.

## Conflict of Interest

The authors declare no conflict of interest.

## Supporting information



Supporting Information

## Data Availability

The data that support the findings of this study are available from the corresponding author upon reasonable request.
