# Supplementary material for: DON‐Loaded Nanodrug‐T Cell Conjugates With PD‐L1 Blockade for Solid Tumor Therapy
Source: Adv Sci (Weinh). 2025 Apr 24;12(26):2501815. doi: 10.1002/advs.202501815 (PMC12245095; doi:10.1002/advs.202501815)
Supplement: Supplementary file 1 — Supporting Information [file ADVS-12-2501815-s001.docx]

DON-Loaded Nanodrug-T Cell Conjugates with PD-L1 Blockade for Solid Tumor Therapy

Xin Yang, Xiaoshuang Niu, Ye Su, Xiaoyun Ye, Wanqiong Li, Wenxuan Zeng, Xin Zhao, Zhuoying He, Qingyu Dong, Xiuman Zhou, Xinghua Sui, Guanyu Chen, Yanfeng Gao*, Juan Liu*

**Affiliations:**

School of Pharmaceutical Sciences (Shenzhen), Shenzhen Campus of Sun Yat-sen University, Shenzhen 518107, China.

***Corresponding authors:**

Prof. Yanfeng Gao and Associate Prof. Juan Liu

School of Pharmaceutical Sciences (Shenzhen), Shenzhen Campus of Sun Yat-sen University, Shenzhen 518107, China.

Tel: +86 755 23260203

E-mail address: [gaoyf29@mail.sysu.edu.cn](mailto:gaoyf@zzu.edu.cn), liuj756@mail.sysu.edu.cn


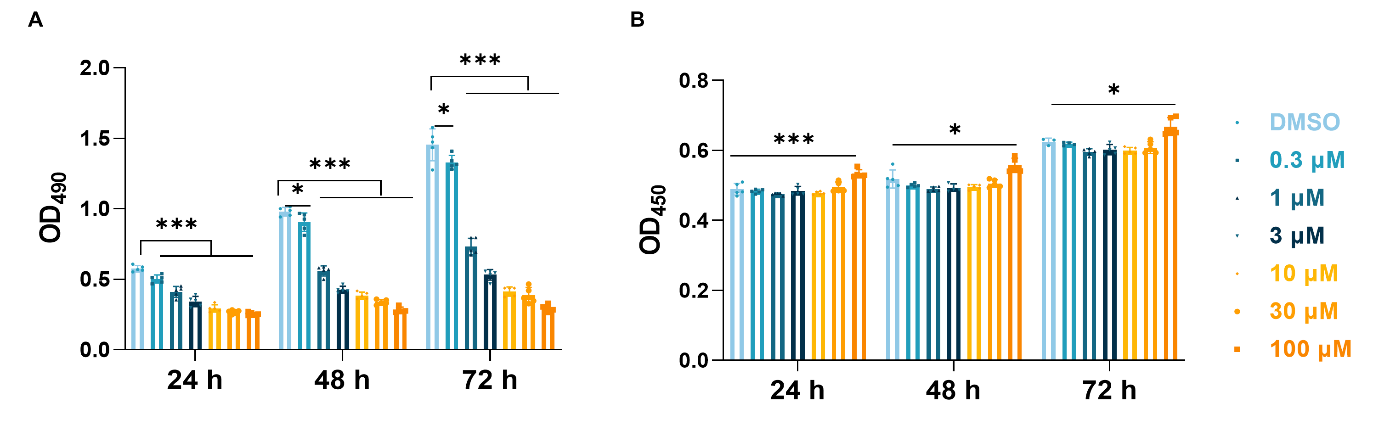


**Figure S1**. Effects of DON on cell proliferation. (A) Cell viability of B16-OVA tumor cells following DON treatment was assessed using the methylthiazolyldiphenyl-tetrazolium bromide (MTT) assay. Cells were seeded in a 96-well plate and incubated overnight, followed by serum starvation for 8 h. Various concentrations of DON were added and co-incubated for 24, 48, and 72 h, and absorbance measurements were taken to evaluate cell viability. (B) Murine-derived CD8^+^ T cells were isolated from the spleens and lymph nodes of C57BL/6 mice, and CD8^+^ T cells were purified using magnetic bead separation. Their activity was assessed using the Cell Counting Kit-8 (CCK-8) assay. n = 3 for all experiments, with statistical significance determined using unpaired, one-sided Mann-Whitney test. **P* < 0.05, ****P* < 0.001.


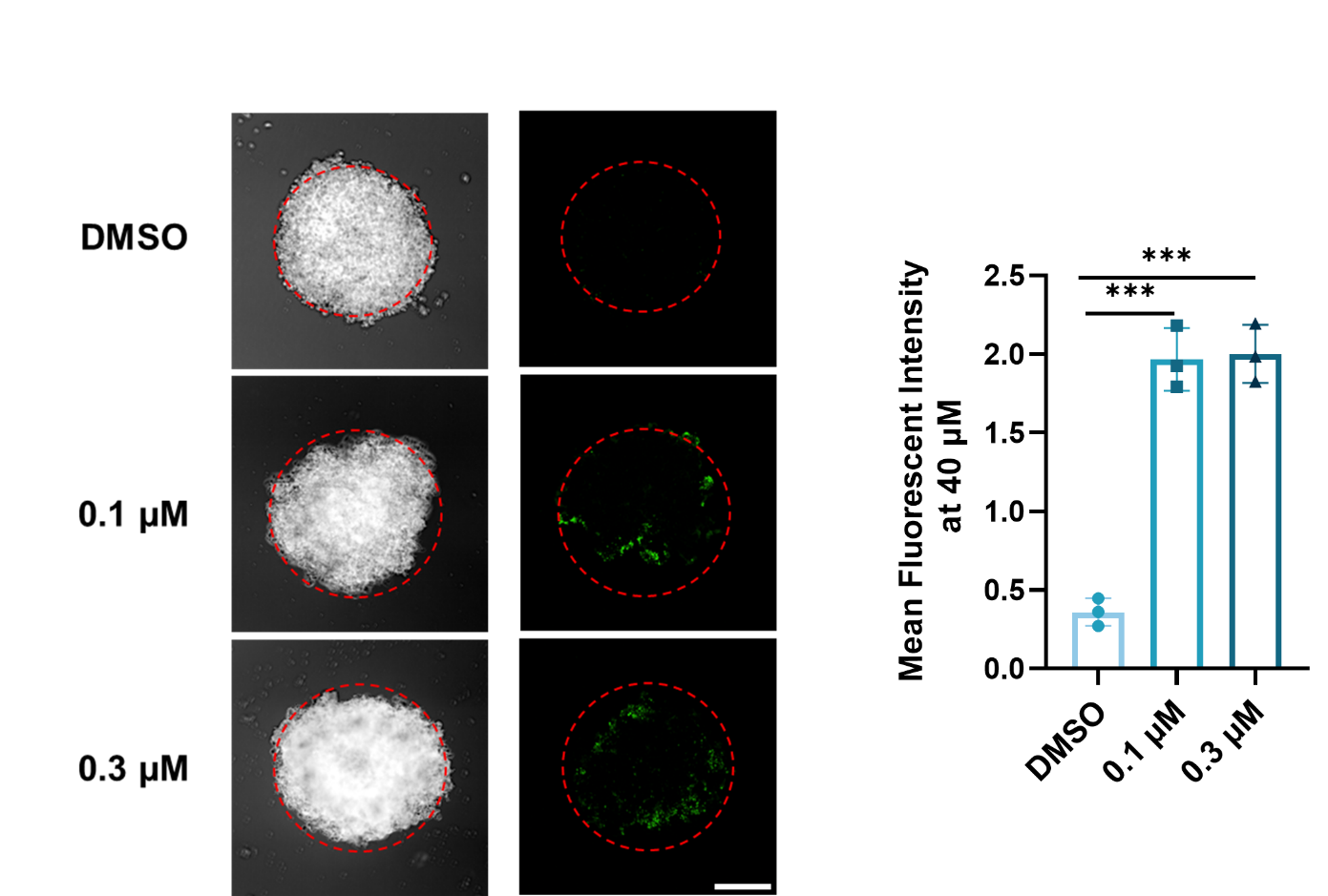


**Figure S2.** DON treatment of B16-OVA spheroids promotes T-cell infiltration. B16-OVA cells were cultured to form dense 3D cell spheroids by centrifugation. On the fourth day after spheroid formation, the spheroids were treated with 0.1 μM or 0.3 μM DON for 24 h. T cells were labeled with carboxyfluorescein succinimidyl ester (CFSE) and co-cultured with spheroids at a density of 1 × 10^6^ cells per well. After 48 h of incubation, the co-cultures were analyzed by confocal microscopy. Scale bar: 200 μm. n = 3 for all experiments, with statistical significance determined using unpaired, one-sided Mann-Whitney test. ****P* < 0.001.


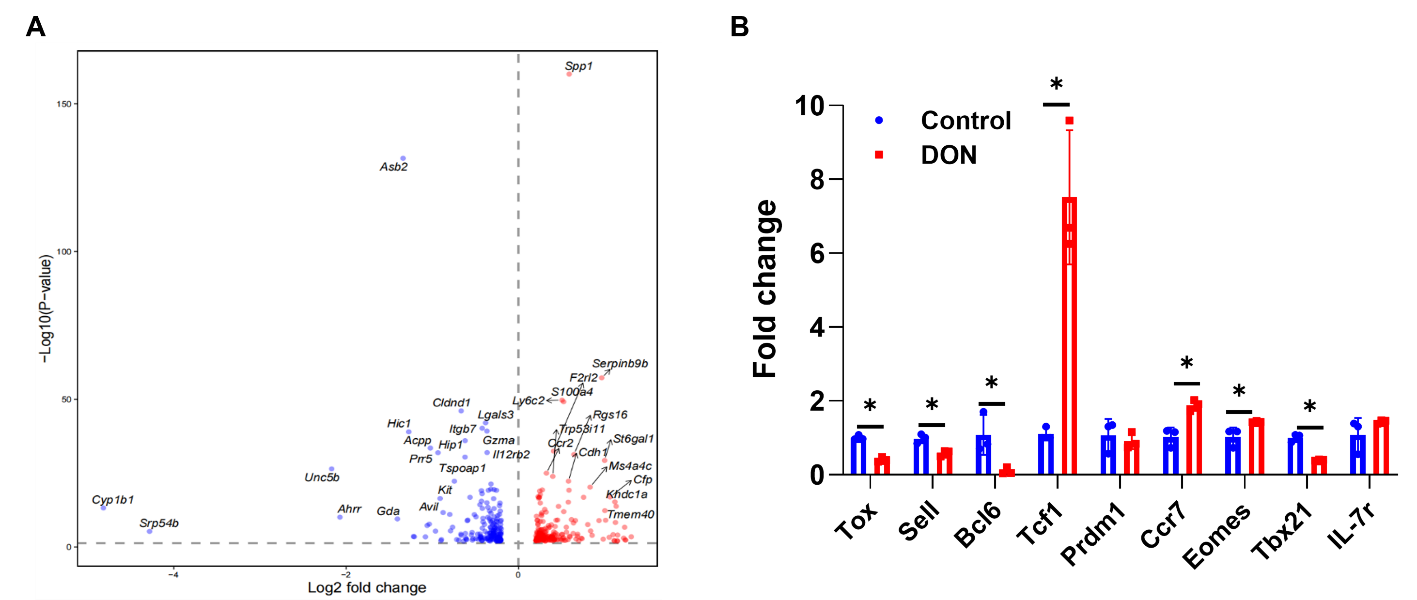


**Figure S3.** DON treatment of B16-OVA spheroids promotes T-cell infiltration. CD8^+^ T cells were sorted from OT-1 mice as described previously and treated with 10 μM DON for 3 days. RNA was extracted for transcriptome analysis (A) and assessed for changes in the expression of stemness-related genes using quantitative polymerase chain reaction (qPCR) (B). n = 3 for all experiments, with statistical significance determined using unpaired, one-sided Mann-Whitney test. **P* < 0.05.


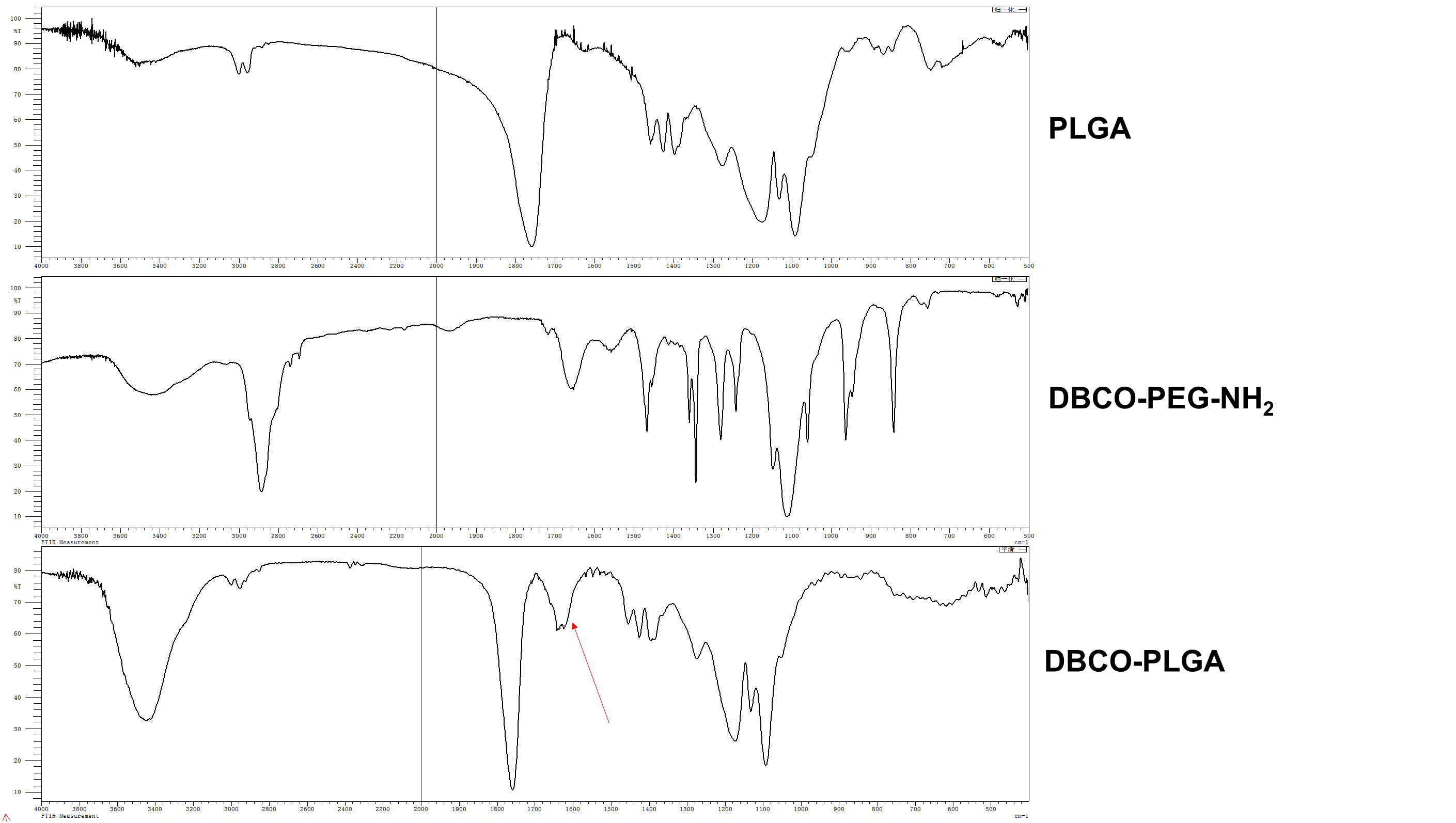


**Figure S4.** Confirmation of dibenzocyclooctyne (DBCO) modification on PLGA nanoparticles by infrared spectroscopy. The coupling group shows a characteristic peak corresponding to DBCO, confirming its presence on the surface of PLGA nanoparticles.


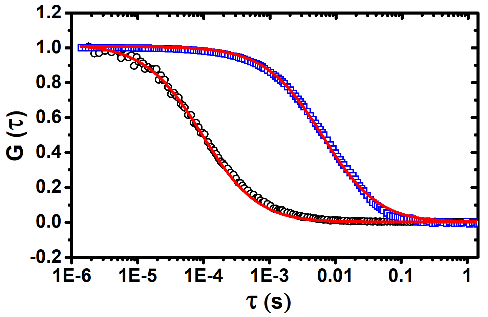


**Figure S5.** Normalized autocorrelation curves from FCS in PBS: Free PLGA (black) and OPBP-1-PLGA (blue). Fluorescence correlation spectroscopy (FCS) data were recorded in PBS for both free atto488-OPBP-1 (black) and OPBP-1-PLGA (blue). The autocorrelation curves were normalized, and the fitting curve based on a two-component model (equation 3) is shown as a red line. The number of peptides per nanoparticle was quantified by dividing the counts per molecule of OPBP-1-PLGA nanoparticles by the counts per molecule of free Atto488-labeled peptides.

**Figure S6.** Optimization of AC_4_ManAz labeling reaction time for murine CD8^+^ T Cells. CD8^+^ T cells were isolated from the spleens and lymph nodes of mice and co-incubated with 50 μM acetylated N-azidoacetylgalactosamine (AC_4_ManNAz) for varying durations. Following this, Cy5-DBCO (dibenzocyclooctyne-conjugated Cy5 dye) was added to assess azide modification efficiency. Cy5 fluorescence (denoted as Cy5^+^) was measured by flow cytometry, and the percentage of Cy5^+^ cells in the CD8^+^ population was calculated. n = 3, with statistical significance determined using a one-way analysis of variance (ANOVA) followed by Tukey's post hoc test for multiple comparisons. ****P* < 0.001.


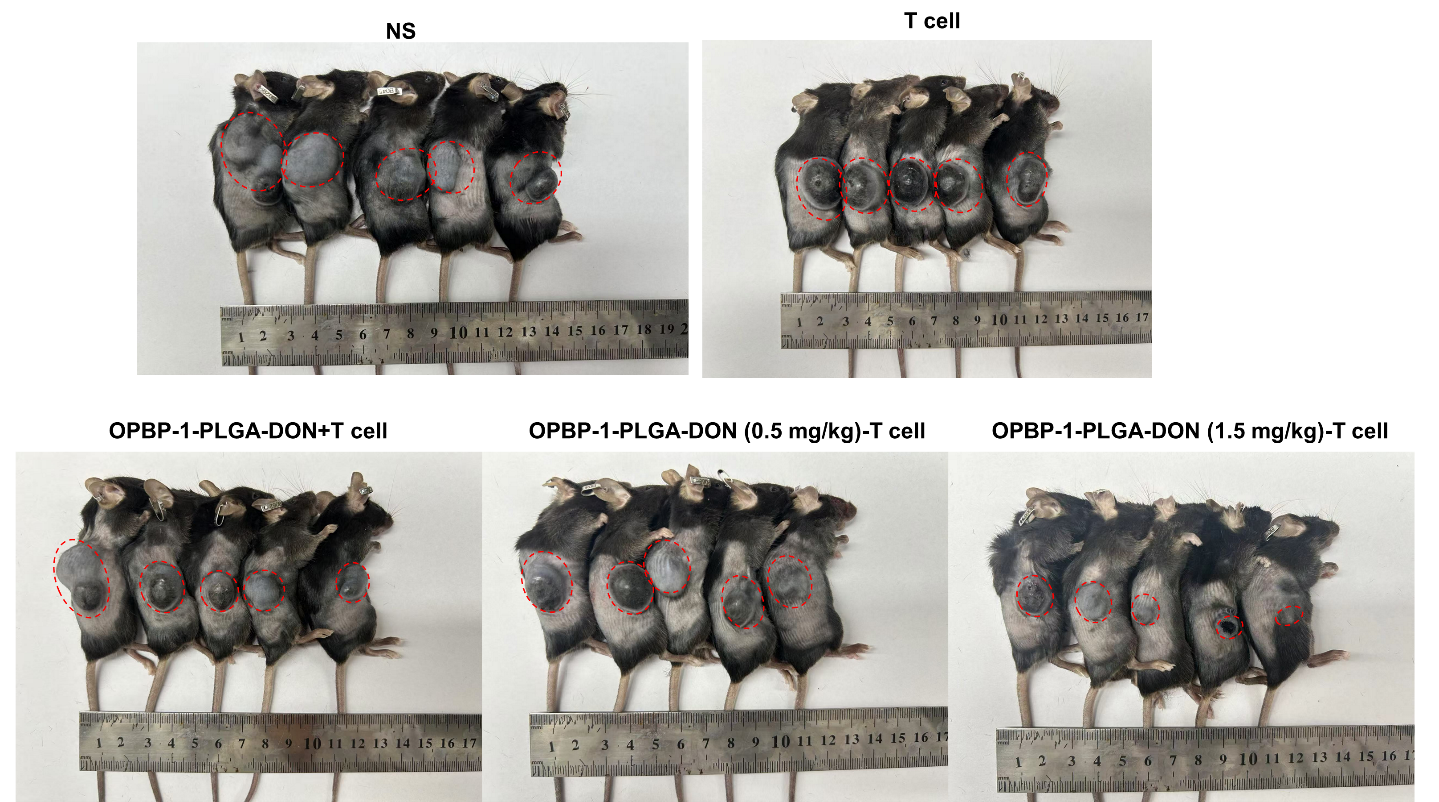


**Figure S7.** Tumor size in B16-OVA model following T cell transfer therapy. B16-OVA cells were cultured, passaged, and prepared into single-cell suspensions, which were then subcutaneously injected into the right dorsal flank of C57BL/6J mice (aged 5-8 weeks). When the tumor volume reached 30-40 mm³ and became relatively uniform, the mice were randomly assigned to different treatment groups. The treatments included physiological saline, T cells, T cells with free OPBP-1-PLGA-DON nanodrugs (OPBP-1-PLGA-DON + T cells), and high and low doses of OPBP-1-PLGA-DON-conjugated T cells (OPBP-1-PLGA-DON-T cells). Mice received tail vein injections of 3×10⁶ T cells on days 9 and 17. n=5.

**Figure S8.** Survival curve of B16-OVA-bearing mice following T cell transfer therapy. The survival curve illustrates the outcomes of B16-OVA-bearing mice following treatment with various regimens. The treatment groups included physiological saline (gray), T cells (blue), T cells with free OPBP-1-PLGA-DON nanodrugs (orange), and OPBP-1-PLGA-DON-T cells at lower (0.5 mg/kg, green) and higher DON doses (1.5 mg/kg, red). n = 12 for each group. Statistical significance between the treatment groups was assessed using the Wilcoxon signed-rank test. ****P* < 0.001.


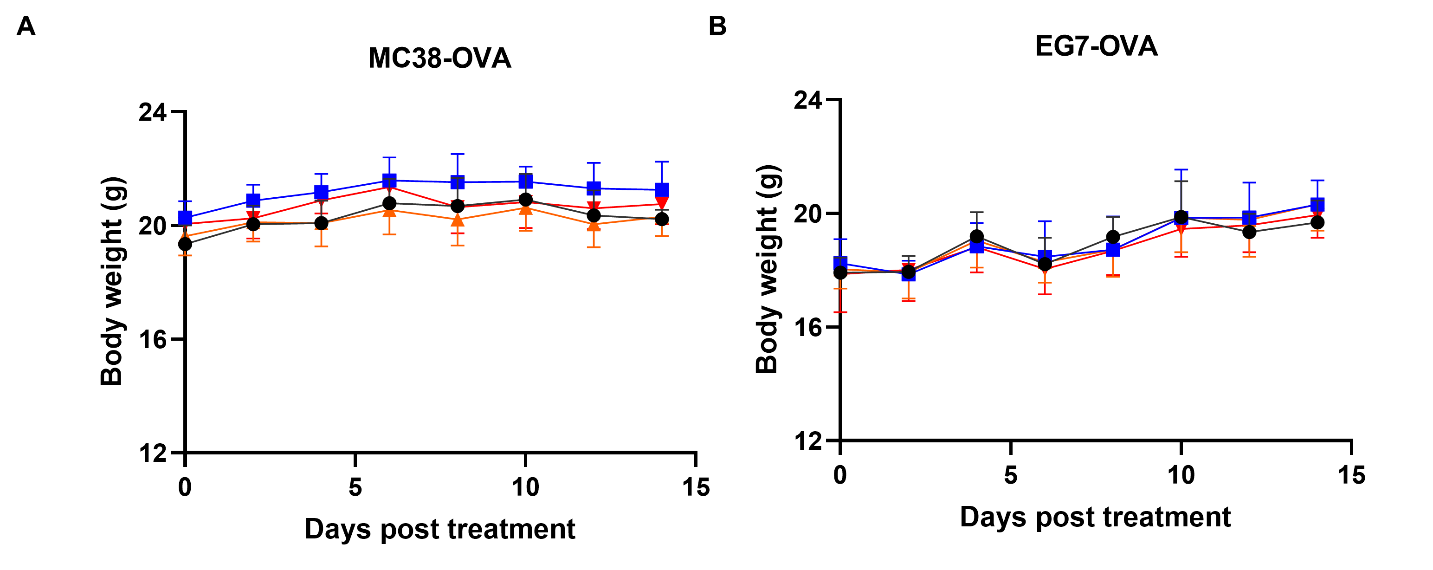


**Figure S9.** Changes in body weight of tumor-bearing mice following T cell transfer therapy. No significant changes in body weight were observed in MC38-OVA tumor-bearing mice (A) and EG7-OVA tumor-bearing mice (B) throughout the treatment period. The treatment groups included physiological saline (black), T cells (blue), T cells with free OPBP-1-PLGA-DON nanodrugs (green), and OPBP-1-PLGA-DON-T cells (red). Mice received tail vein injections of 3×10⁶ T cells on days 7 and 14. n = 5 for each group. Statistical significance was assessed using a one-way ANOVA followed by Tukey's post hoc test for multiple comparisons.


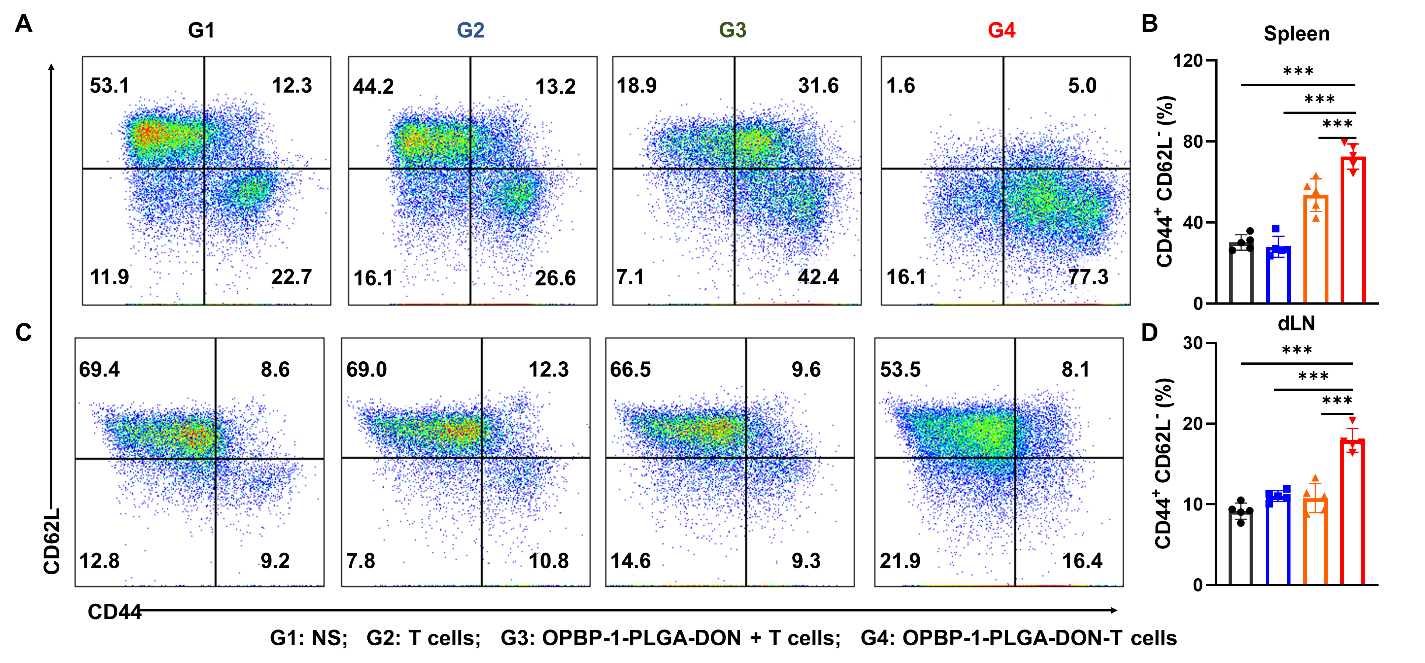


**Figure S10.** Proportion of effector memory T (TEM) cells in CD4^+^ T cells of MC38-OVA tumor-bearing mice following T cell transfer therapy. Lymphocytes were isolated from the spleens and draining lymph nodes (DLN) of tumor-bearing mice. CD44 and CD62L expression in CD4^+^ T cells was analyzed by flow cytometry and statistically assessed (A,B for spleen, C,D for DLN). Mice received 3×10⁶ T cells via tail vein injections on days 7 and 14. Treatment groups included physiological saline (G1), T cells (G2), T cells with free OPBP-1-PLGA-DON nanodrugs (G3), and OPBP-1-PLGA-DON-T cells (G4). n = 5 for all experiments, with statistical significance determined using a one-way ANOVA followed by Tukey's post hoc test for multiple comparisons. ****P* < 0.001.
